# Integrated single-cell transcriptomics and proteomics elucidate the molecular mechanisms and detoxification strategy of rifampicin-induced hepatotoxicity

**DOI:** 10.7150/ijbs.109757

**Published:** 2026-01-01

**Authors:** Junhui Chen, Qian Zhang, Jingnan Huang, Hengkai He, Yunmeng Bai, Yehai An, Mingjing Hao, Wentong Zhao, Wenhui Li, Sha Feng, Shiguang Yang, Jiaxian Liao, Yin Kwan Wong, Lingyun Dai, Qingfeng Du, Piao Luo, Jigang Wang

**Affiliations:** 1 Department of Pulmonary and Critical Care Medicine, Shenzhen Institute of Respiratory Diseases, Guangdong Provincial Clinical Research Center for Geriatrics, Shenzhen Clinical Research Center for Geriatrics, Shenzhen People's Hospital, The First Affiliated Hospital, School of Medicine, Southern University of Science and Technology, Shenzhen, 518055, China.; 2 Guangdong Basic Research center of Excellence for Integrated Traditional and Western Medicine for Qingzhi Diseases, Guangdong provincial Key Laboratory of Chinese Medicine pharmaceutics, School of Traditional Chinese Medicine and School of pharmaceutical Sciences, Southern Medical University, Guangzhou 510515, Guangdong, China.; 3 School of Traditional Chinese Medicine and School of Pharmaceutical Sciences, Southern Medical University, Guangzhou 510515, Guangdong, China.; 4 Institute of Acupuncture and Moxibustion, Shandong University of Traditional Chinese Medicine, 4655 University Road, Jinan 250355, China.

**Keywords:** Rifampin, Hepatocytes, Reactive oxygen species, Oxidative stress, Rosmarinic acid.

## Abstract

Rifampicin (RIF), a cornerstone drug in tuberculosis treatment, is associated with hepatotoxicity, which represents a significant adverse effect that frequently causes discontinuation of therapy. However, a comprehensive evaluation of the mechanisms underlying RIF-induced hepatotoxicity remains limited, and the identification of highly effective, low-toxicity therapeutic interventions is urgently needed. In this study, we employed a RIF-induced mouse hepatotoxicity model to systematically investigate the cellular and molecular events associated with RIF-induced liver injury. By integrating single-cell RNA sequencing, bulk RNA-seq, and mass spectrometry-based proteomics and metabolomics, we identified region-specific hepatocyte damage characterized by elevated reactive oxygen species (ROS) levels and activation of the fatty acid oxidation pathway. At the molecular level, RIF treatment resulted in the upregulation of pregnane X receptor (PXR) and *Cyp3a11*, along with the downregulation of key antioxidant genes. Moreover, decreased mTOR expression and increased expression of fatty acid oxidation-related genes including *Acox1* and *Acaa1b* suggested an enhanced oxidative metabolism. Recruitment of macrophages further exacerbated hepatocyte damage. Importantly, *Rosmarinic acid* (RA) administration was shown to attenuate RIF-induced hepatotoxicity. These findings provide a comprehensive molecular and cellular perspective on RIF-induced hepatotoxicity and suggest the potential clinical application of RA as a therapeutic agent in the management of RIF-induced liver injury.

## Introduction

Tuberculosis (TB) is caused by the *Mycobacterium tuberculosis* complex, which has long posed a great threat to human health. Basing on the Global Tuberculosis Report 2022, ~10.6 million people were infected with TB in 2021, with the incidence rate rising by 3-6% compared to 2020 1. First-line anti-tuberculosis drugs such as isoniazid, rifampicin, pyrazinamide, and ethambutol are widely used, but three of these (isoniazid, rifampicin, and pyrazinamide) have the potential to cause hepatotoxicity 2, 3. Hepatotoxicity is a significant side effect of TB treatment in clinical practice, often causing the discontinuation of medication. Studies have linked the mechanism of liver damage to oxidative stress induced by the metabolites of these drugs, resulting in excessive ROS that damage DNA and oxidize lipids and proteins in hepatocytes, thereby contributing to hepatotoxicity 4, 5.

RIF, approved by the Food and Drug Administration (FDA) for TB treatment in 1971, remains a cornerstone of TB pharmacotherapy 6. Its use has expanded significantly as part of combination antimicrobial therapies for various infections 7, 8. As one of the most potent known inducers of metabolic enzymes, RIF strongly activates the PXR, leading to increased expression of drug-metabolizing enzymes including human *CYP3A4* (murine homolog *Cyp3A11*) 9-11. Consequently, when used in combination with other anti-TB drugs such as isoniazid, RIF can increase the production of toxic intermediate metabolites by inducing *CYP2E1*, thereby exacerbating isoniazid-induced hepatotoxicity 12. RIF itself can be metabolized by cytochrome P450 enzymes into toxic intermediates 13, and the accumulation of these intermediates induces excessive ROS production, causing lipid peroxidation and further damage to hepatocytes 14-16.

Currently, there are no suitable drugs available to effectively treat hepatotoxicity caused by anti-tuberculosis drugs, highlighting the urgent need for new therapies that combine high efficacy with low toxicity. RA, a natural phenolic compound, acts as a scavenger of ROS, inhibits lipid peroxidation and exhibits anti-inflammatory properties 17. Studies suggest that RA improves drug-induced liver injury and demonstrates hepatoprotective effects 18, 19. However, to our knowledge, the mechanisms underlying RIF-induced hepatotoxicity have not yet been fully elucidated, and the potential of RA to treat hepatotoxicity induced by anti-tuberculosis drugs has not been explored.

Multi-omics technologies offer unique advantages for comprehensive analysis of drug-induced toxicity, and recent developments in scRNA-seq have facilitated toxicology research at the resolution of individual cells 20-22. Herein, we constructed a mouse model of RIF-induced hepatotoxicity and employed bulk RNA-seq, scRNA-seq, proteomics and metabolomics to comprehensively analyze the mechanisms of RIF-induced hepatotoxicity. Our results show that RIF induces localized damage to hepatocytes by activating PXR and *Cyp3a11*, which is accompanied by increased ROS production, oxidative stress, and the activation of fatty acid oxidation. The recruitment of macrophages aggravates hepatocyte injury. Subsequently, we investigated the potential of RA to ameliorate RIF-induced hepatotoxicity. The findings offer a comprehensive understanding of RIF-induced hepatotoxicity and suggest that RA may reduce ROS production and apoptosis, thereby alleviating liver damage. Collectively, the study provides clinical insights into the mechanisms of RIF-induced hepatotoxicity and potential therapeutic interventions.

## Materials and Methods

### Materials and reagents

Rifampin (RIF, purity > 98.0%) was obtained from TCI Shanghai. The Cell Counting Kit-8 (CCK-8) was sourced from Dojindo (Kyushu, Japan). ALT, AST, ALP, BILD, BILT and TBA assay kits were acquired from Bejian Xinchuangyuan Biotech (Beijing, China). Primary antibodies, including anti-TNF-α (Cat# 17590-1-AP), β-actin (Cat# 66009-1-Ig), anti-PXR (Cat# 67912-1-Ig), anti-Bax (Cat# 50599-2-lg), anti-Bcl-2 (Cat# 12789-1-AP) and anti-Cleaved Caspase-3 (Cat# 25128-1-AP) were purchased from Proteintech (Chicago, USA).

### Cell cultivation and treatment

The mouse normal hepatocyte line (AML12) was cultured in DMEM supplemented with 10% fetal bovine serum, penicillin and streptomycin (Gibco, Foster, CA, USA) under controlled environmental conditions. The cells were treated with RIF for either 24 or 48 hours, after which they were harvested for western blot analysis.

### Animals and rifampin treatment

C57BL/6 mice (6-8 weeks old, weighing 21±2g) were obtained from GemPharmatech (Guangdong, China). The mice were housed in specific pathogen-free (SPF) environment under suitable temperature and humidity. The mice were randomly assigned to two groups: a control group (Ctrl) and a rifampicin-treated group (RIF). Mice in the RIF group received an injection of RIF once daily for three weeks. Meanwhile, Control mice received an equal amount of vehicle. Following the treatment period, the mice were anesthetized, followed by the collection of blood samples and liver tissues.

### Serum biochemistry and histopathology

Serum levels of ALT, AST, ALP, BILD, BILT and TBA were measured using an automatic biochemistry analyzer (TOSHIBA, Japan). Liver tissues were paraffin-embedded, sectioned and histological changes were evaluated through H&E staining, following the methods outlined in our previous study 23.

### Western blotting analysis

Protein samples were lysed using immunoprecipitation lysis buffer supplemented with 1× protease inhibitor. The concentration of extracted proteins was measured using a bicinchoninic acid (BCA) assay kit (Thermo Fisher, USA). Equal amounts of protein from each sample were separated by sodium dodecyl sulfate-polyacrylamide gel electrophoresis (SDS-PAGE) and transferred onto polyvinylidene fluoride (PVDF) membranes. After blocking, Overnight incubation of membranes with primary antibodies was performed at 4°C (anti-TNF-α, anti-PXR, anti-β-actin, anti-Bax, anti-Bcl-2, and anti-Cleaved Caspase-3) and then with appropriate secondary antibodies. Protein bands were detected using an Azure Sapphire imaging system, and intensities were semi-quantified using ImageJ.

### Apoptosis flow cytometry

Apoptosis was assessed using an apoptosis detection kit (BD Biosciences, USA). The apoptosis rate was assessed by counting the number of cells stained positively with Annexin V and propidium iodide. After washing, the cells treated with RIF, DMSO, or RIF+RA were transferred to a binding buffer containing the dyes, incubated at room temperature for 15 minutes, followed by flow cytometric analysis within one hour.

### ROS flow cytometry

ROS levels were measured using the ROS Assay Kit (Jiancheng Bioengineering Institute, Nanjing, China). Cells treated with RIF, DMSO, or a combination of RIF and RA were washed and stained with dye-containing binding buffer (15 min, RT). Flow cytometric analysis was performed on all samples within 60 minutes.

### Quantity real-time PCR

Quantitative real-time PCR was performed to validate mRNA expression differences in hepatic cells treated with RIF, DMSO, or a combination of RIF and RA. Cells were isolated using TRIzol following the manufacturer's guidelines. Subsequently, after reverse transcription, the expression of the relevant mRNAs was assessed using RT-qPCR (Hieff qPCR SYBR Green Master Mix, YEASEN, China). The primer sequence for *Cyp3a11* is provided in Table S5.

### Single-cell suspensions

Mice from both the Ctrl and RIF groups were selected. Fresh liver tissues were collected from 3 control mice and 3 RIF-treated mice, which were then cut into small pieces. The liver fragments were digested following the manufacturer's protocol (Miltenyi Biotec). After digestion, the samples were filtered, centrifuged and resuspended. Red blood cells were removed, followed by two washes with phosphate-buffered saline (PBS).

### scRNA-seq

Basing on the 10x Genomics official protocol, single-cell suspensions were utilized to prepare sequencing libraries using the Single Cell 3′ Reagent Kit v3.1 (10x Genomics). The prepared libraries were subsequently sequenced on Illumina NovaSeq 6000 platform.

### scRNA-seq data quality control and processing

The raw scRNA-seq reads was filtered with fastp (version 0.20.0) to eliminate low-quality reads 24. High-quality reads were then processed with the Cellranger pipeline (version 7.0.0) to generate expression matrices for each sample. Subsequent filtering of low-quality cells was performed using the Seurat package 25, retaining only cells that met the criteria as Table S4 provided. To facilitate downstream analyses, the data from all samples were integrated into a normalized, unbatched dataset using Seurat. This integrated dataset underwent PCA and UMAP for dimensionality reduction. Cells was clustered using Seurat's FindClusters function with a resolution parameter of 1.2. The identity of each cluster was determined by combining canonical markers with cluster-specific markers.

### Differential gene expression and pathway analysis

DEGs for each cell type were identified by Seurat with default parameters. Genes meeting the criteria of |log_2_FC| ≥ 0.25 and an adjusted *p*-value < 0.05 (where FC represents fold change) were considered as DEGs. When analyzing gene regulatory relationships in Hep2, |log_2_FC| ≥ 0.1 was used as the threshold. Functional enrichment analysis of the DEGs was performed using the clusterProfiler package (version 4.0.0), and *p* value was adjusted using the Benjamini-Hochberg method 26. Additionally, gene set enrichment analysis was completed using the gseGO and gseKEGG functions of clusterProfiler. The results of the gene set analysis were visualized with the gseaplot2 function, a module of the enrichplot R package.

### Pseudotime analysis

Pseudotemporal analysis and cell fate inference were conducted using the Monocle2 R package 27.

### Cell-cell intercellular networks

Cellular crosstalk between hepatocytes and immune cells was systematically analyzed based on ligand-receptor interactions using the CellChat package 28. Specifically, the functions netVisual_aggregate and netVisual_chord_gene were employed to assess the interactions between hepatocytes and macrophages.

### Construction and analysis of gene regulatory networks

Gene regulatory network analysis in hepatocytes was conducted using the PySCENIC package. The regulatory activities of transcription factors were visualized with the pheatmap function.

### Bulk RNA-Seq and data analysis

RNA was isolated from liver samples (three control and three RIF-treated) using the Qiagen RNeasy Mini Kit and enriched via poly(A) sorting. RNA-seq was performed subsequently on the Illumina NovaSeq 6000 platform (Illumina) with paired-end 150 (PE150) reads. Raw reads were filtered using fastp. The filtered reads were subsequently aligned to the mouse reference genome (version mm10) by HISAT2 (version 2.1.0) 29. FeatureCounts (version 2.0.1) was used to carry out gene expression quantification. Normalization of gene expression and identification of DEGs were carried out using edgeR (version 3.34.0) 30. DEGs were defined as genes with |log_2_ fold change| ≥ 1 and an adjusted *p*-value (FDR) < 0.05. For the scRNA-seq dataset, the AggregateExpression function in Seurat was used to aggregate counts for each sample, generating bulk RNA data. DEGs in these bulk samples were then identified using FindMarkers.

### Proteomics analysis

Liver tissue was lysed with RIPA lysis buffer and subjected to sonication on ice. Following centrifugation for 15 minutes at 4°C, the supernatant was subjected to reduction and alkylation. The sample was then precipitated with pre-cooled acetone at -20°C for over 1 hour and centrifuged for 15 minutes at 4°C. The precipitate was dried and subsequently solubilized in 8 M urea/100 mM TEAB lysis buffer (pH 8.5). Protein samples were digested overnight at 37°C with trypsin (12.5 ng/μl) and CaCl2 (1 mM), and centrifuged at 12,000 × g for 5 min at 37°C, and the resulting supernatant was subjected to C18 desalting. The column was first equilibrated with 0.1% formic acid wash buffer, followed by elution using 60% acetonitrile/0.1% formic acid solution. The eluate was collected, lyophilized and analyzed by LC-MS/MS using a Thermo Orbitrap Fusion Lumos (USA). Mass spectrometry raw data were processed using Proteome Discoverer 2.4 (Thermo Fisher Scientific), with parameter settings detailed in our previous study 31. Subsequent analysis of the proteomic data was performed using the DEP R package, which included missing value imputation, data normalization, and differential expression analysis. Differentially expressed proteins were defined as those meeting the threshold criteria of *p* value < 0.05 and absolute fold change > 1.2. Biological process enrichment analysis of these differentially expressed proteins was carried out using the clusterProfiler package, with parameter settings aligned with those used for bulk RNA-seq analysis 26. Differentially expressed proteins were visualized using the R packages ggplot2 and pheatmap.

### Metabolomic analysis

Liver sample preparation and metabolite extraction were conducted following established protocols 32. The extracted metabolite samples were analyzed using LC-MS/MS with a Thermo Orbitrap Fusion Lumos (USA). Raw metabolite data were preprocessed using Compound Discoverer 3.1 (CD3.1), which integrates high-quality mzCloud, mzVault, and MassList databases for component identification. CD3.1 also performed relative quantification based on the area of characteristic peaks. Metabolites detected in both ion modes were merged, and PCA and orthogonal partial least squares discriminant analysis (OPLS-DA) were conducted using the ropls package (version 1.30.0) in R 33. Subsequent variance analysis of the detected metabolites was performed using t-tests, with multiple hypothesis testing corrected using the p.adjust function in R. Differential metabolites were defined as those with an *p*-value < 0.05, absolute fold change > 1.2, and a Variable Importance in Projection (VIP) score greater than 1. KEGG enrichment analysis of these differential metabolites was carried out using MetaboAnalyst 6.0 34.

### Data availability

The sequencing raw data for scRNA-seq have been deposited in OMIX under accession number OMIX007370, while the bulk RNA-seq data have been deposited in the Genome Sequence Archive (GSA) with accession number CRA018977. Additional data supporting the conclusion are provided in the supplementary materials.

### Statistical analysis

Data are expressed as mean ± SD from three or more independent biological replicates. Statistical analyses were carried out using GraphPad Prism 8.0 and R software. Schematic diagrams were created using BioRender.com. Comparisons between two groups were made using an unpaired two-tailed t-test, unless otherwise specified. Kruskal-Wallis test or one-way ANOVA for comparison of multiple groups.

## Results

### RIF triggers liver toxicity and dysfunction in mice

**Figure 1:**
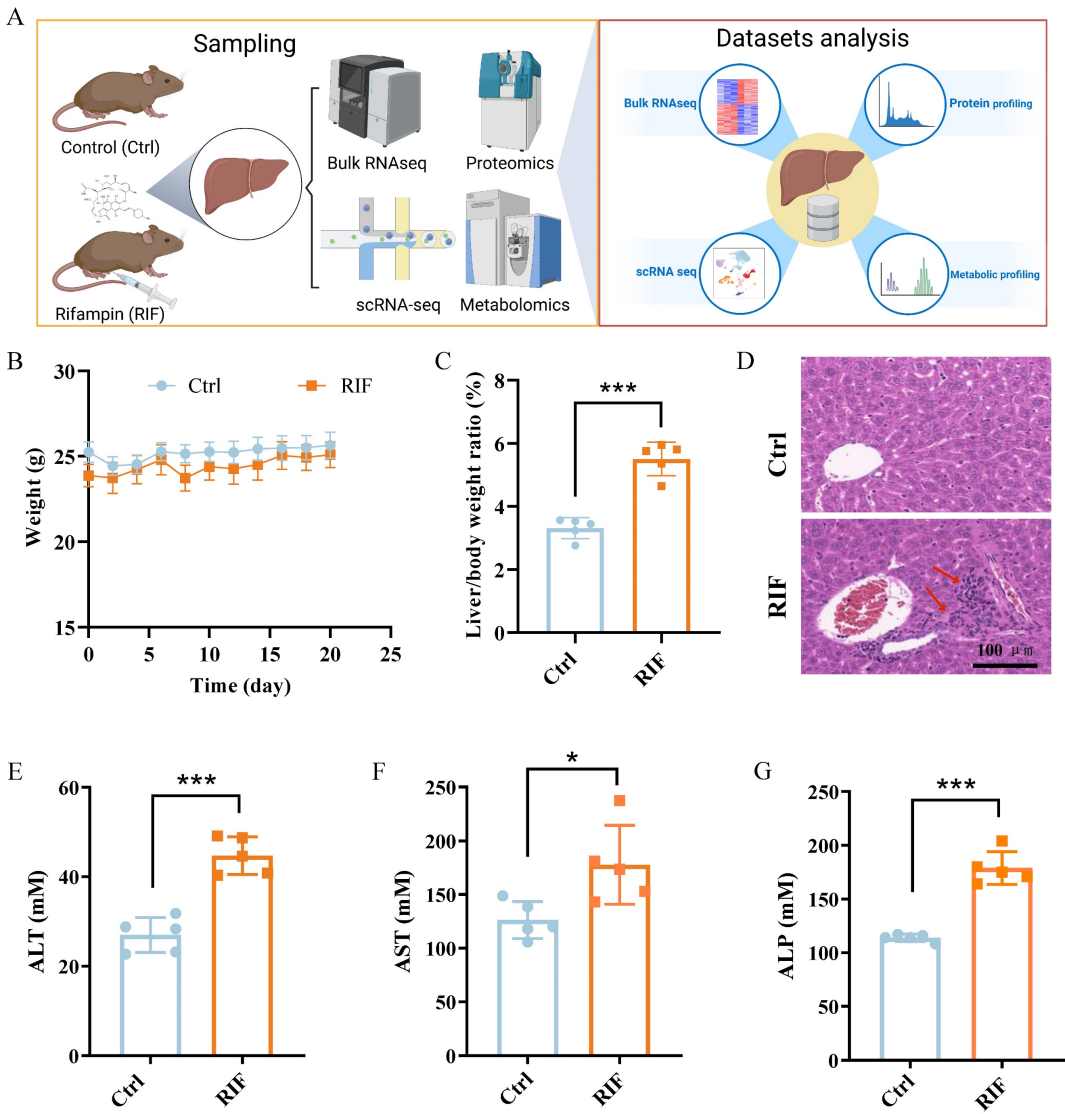
RIF triggered liver toxicity and dysfunction in mice**.** (A) The scheme of the study design. (B) Weight changes of mice in control and RIF groups. (C) The liver/body weight ratio in two groups. (D) H&E staining of control and RIF exposure liver (scale bar: 100 μm; Red arrow indicating inflammatory cell infiltration). (E-G) Levels of serum ALT, AST and ALP in Ctrl and RIF groups (n=5, * *p* < 0.05, *** *p* < 0.001).

To investigate RIF-induced hepatotoxicity, C57BL/6 mice were treated with RIF. Samples were collected following RIF administration and underwent scRNA-seq, bulk RNA-seq, mass spectrometry-based protein profiling and comprehensive metabolic profiling. The experimental workflow is depicted in Figure 1A. Firstly, the weight and biochemical parameters of mice following RIF exposure were assessed. Compared to the control groups, body weight slightly decreased, while the liver-to-body weight ratio significantly increased following RIF treatment (Figure 1B-C). H&E staining revealed inflammatory cell infiltration, indicating liver injury and morphological alterations (Figure 1D). Notably, key liver function enzymes—alanine aminotransferase (ALT), aspartate aminotransferase (AST), and serum alkaline phosphatase (ALP) showed significant increases post-RIF treatment (Figure 1E-G). Bilirubin is a well-established marker for biochemical tests for patients with liver dysfunction, and serum levels of direct bilirubin (BILD), total bilirubin (BILT), and total bile acid (TBA) were elevated in the RIF-treated group (Figure S1A-C). Collectively, these findings suggest that RIF induces hepatotoxicity and liver dysfunction.

### RIF-induced hepatotoxicity involving metabolism dysfunction

**Figure 2:**
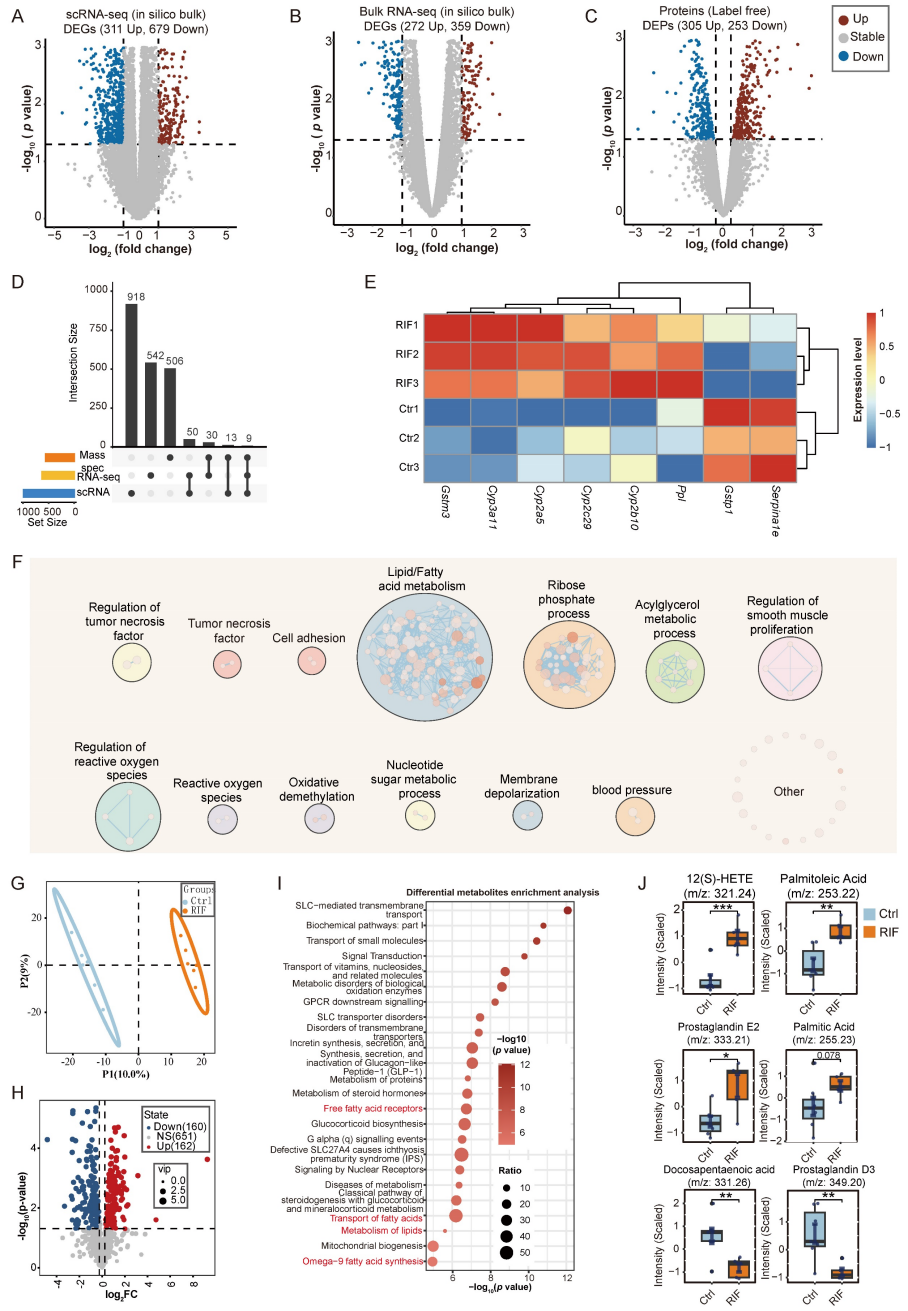
RIF-induced hepatotoxicity involving metabolism dysfunction. (A-C) Volcano plot displays DEGs in pseudo bulk data of scRNA, bulk RNA-seq and DEPs in Ctrl *vs.* RIF, respectively. (D) The UpSet plot shows the relationship of shared altered genes upon RIF across three datasets. (E) Expression level of common altered genes in the datasets*.* (F) Biological process (BP) clusters of the genes that exhibit consistent changes across the mRNA datasets (scRNA-seq and RNA-seq) and the mass spectrometry data. (G) Ortho PLS-DA analysis of metabolites in the Ctrl and RIF groups in the liver. (H) Volcano plot shows distribution of differential metabolites. (I) KEGG enrichment of differential metabolites. (J) Detecting intensity of top differential metabolites (ranked by VIP) in two groups (n=6, * *p* < 0.05, ** p < 0.01, *** *p* < 0.001).

For comparative analysis of gene expression across multi-omics datasets, scRNA-seq counts were aggregated by sample conditions using Seurat's AggregateExpression function. Subsequently, we identified genes and proteins exhibiting significant changes in response to RIF treatment across the datasets. Specifically, analysis of the scRNA-seq data revealed 990 DEGs (311 upregulated; 679 downregulated) (Figure 2A), while the bulk RNA-seq data identified 631 DEGs (272 upregulated; 359 downregulated) (Figure 2B; Table S1). In the mass spectrometry dataset, we quantified 5,318 proteins, of which 558 were differentially expressed (305 upregulated; 253 downregulated) (Figure 2C; Table S2). Strong correlations in expression changes were observed across the three omics datasets (R > 0.61, *p* < 0.05) (Figure S2A-C). Analysis of overlapping expression changes revealed 52 genes with consistent alterations in both mRNA and protein datasets in response to RIF treatment (Figure 2D). Notably, *Cyp3a11, Cyp2a5, Cyp2c29* and *Cyp2b10,* which are involved in drug metabolism, were commonly upregulated across all three omics datasets, consistent with the activation of P450 enzymes by RIF (Figure 2E).

To further investigate the shared alterations between mRNA and protein datasets, we performed enrichment analysis on the genes that exhibited consistent changes across the mRNA datasets (scRNA-seq and RNA-seq) and the mass spectrometry data. The top 5 results revealed significant enrichment in metabolic processes, such as fatty acid metabolic and glutathione metabolic process (Figure S2D; Table S3). Then, we visualized all gene-set enrichment results as a network using EnrichmentMap, followed by identifying clusters of nodes with AutoAnnotate. The largest cluster was associated with lipid/fatty acid metabolism, followed by clusters related to ribose phosphate processes and acylglycerol metabolism. Pathways involved in TNF signaling and cell adhesion were also prominent, alongside processes linked to ROS (Figure 2F).

Next, we performed untargeted metabolomics on liver tissue from control and RIF-induced mice. Using the UHPLC-MS/MS technique, we detected 973 metabolites in both positive and negative ion modes. Quality control samples demonstrated a high correlation coefficient (Figure S2E), and PCA showed complete separation of the quality control samples from both control and RIF-induced groups (Figure S2F-G), suggesting that the detection process is stable and of high quality in both ion modes. Ortho PLS-DA further confirmed clear separation between the control and RIF groups (Figure 2G). Together, these findings indicate that the classification model is both stable and dependable. Subsequently, we combined data from both ion modes to identify differential metabolites in the liver upon RIF treatment. The expression patterns of these differential metabolites are shown in Figure S2H. In total, 162 upregulated and 160 downregulated metabolites were identified (*p*-value < 0.05 and VIP > 1) (Figure 2H). Differential metabolites were annotated based on the Human Metabolome Database (HMDB) classification, revealing that most altered metabolites were carboxylic acids and derivatives (26.08%) and fatty acyls (19.86%) (Figure S2I). We then conducted KEGG enrichment analysis on the differential metabolites. Results suggest that RIF may affect omega-9 fatty acid synthesis, mitochondrial biogenesis, fatty acid transport and lipid metabolism (Figure 2I). In detail, we ranked the differential metabolites related to lipid metabolism based on their VIP scores in decreasing order, highlighting the top metabolites such as 12(S)-HETE, palmitoleic acid, palmitic acid, prostaglandin E2, docosapentaenoic acid and prostaglandin D3 (Figure 2J).

Collectively, these results suggest that RIF-induced hepatotoxicity is mediated by disruptions in metabolic processes, particularly lipid metabolism.

### Single cell transcriptomic profiling reveals heterogeneous changes upon RIF exposure

**Figure 3:**
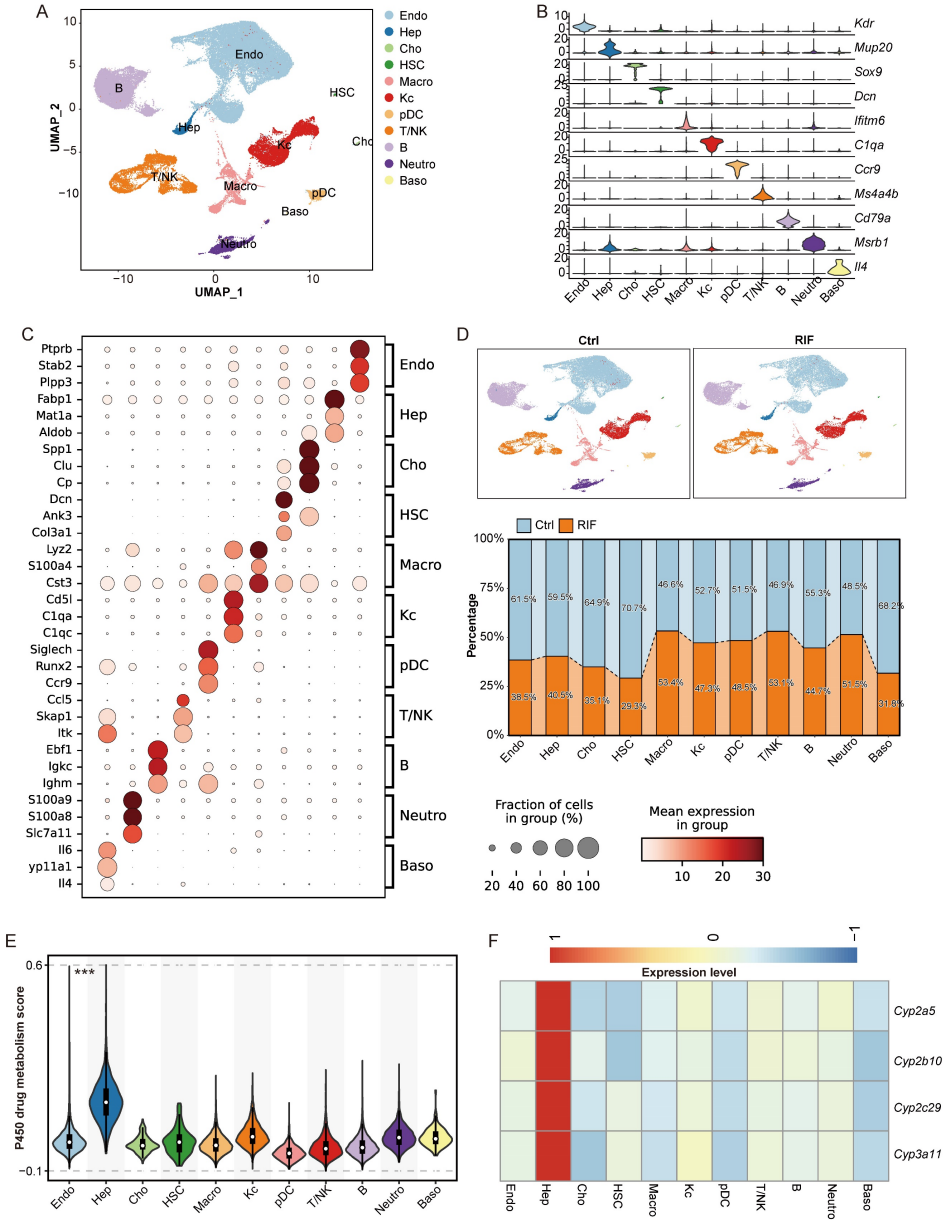
Single cell transcriptomic profiling revealing heterogeneous changes upon RIF exposure. (A) UMAP track displays 11 cell types basing on 67,447 single-cell transcriptomes in mice liver. (B) Expression levels of canonical markers in 11 cell types. (C) Dot plot shows the top 3 marker genes in each cell types. (D) Cellular proportion of cell types in control and RIF groups. (E) P450 drug metabolism gene set score in each cell type (*** *p* < 0.001). (F) The relative expression of genes associated with P450 drug metabolism in each cell type (these genes displaying a consistent upregulation in the omics data).

The liver possesses a complex architecture and diverse cellular composition, including hepatocytes, hepatic stellate cells, endothelial cells, immune cells such as T cells, B cells and macrophage. Cellular diversity contributes to significant variability in drug metabolism and drug-induced hepatotoxicity across different cell types and specific hepatic zones. To investigate RIF-induced hepatotoxicity with greater precision, we employed scRNA-seq to analyze liver tissues from mice exposed to RIF. We initially isolated 84,897 cells from the livers of control and RIF-treated mice. After quality control, 67,447 cells (37,767 from control and 29,680 from RIF treatment) were retained for subsequent analysis (Figure S3A; Table S4). These cells were integrated into a normalized and unbatched dataset. We identified 44 distinct clusters using the dataset by Seurat (Figure S3B). By combining classical markers with cluster-specific markers, we annotated these clusters into 11 cell types: endothelial cells (Endo), hepatocytes (Hep), cholangiocytes (Cho), hepatic stellate cells (HSC), macrophages (Macro), Kupffer cells (Kc), plasmacytoid dendritic cells (pDC), T/NK cells (T/NK), B cells (B), neutrophils (Neutro), and basophils (Baso) (Figure 3A).

The expression of classical markers was consistent with the identified cell types (Figure 3B; Figure S3C). The identity of each cell type was further validated by the top three marker genes. For instance, *Ptprb* and *Stab2* were predominantly expressed in Endo; *Fabp1* and *Mat1a* were specific to Hep; *Lyz2* and *S100a4* were highly expressed in Macro; and *C1qa* and *C1qc* were expressed in Kc (Figure 3C; Table S6). These expression profiles align with those recorded in the Panglao database, supporting the accuracy of our cell type annotations.

Upon comparing the control and RIF-treated groups, we observed a significant reduction in the proportion of Hep following RIF exposure, while the relative abundance of leukocytes including T/NK cells, macrophages, and neutrophils increased (Figure 3D; Figure S3D-E). The decrease in Hep aligns with the known hepatotoxic effects of RIF 35. Other cell types exhibited variable reductions post-RIF exposure. Additionally, P450-related metabolic enzymes are extensively involved in drug metabolism, generating active metabolites that contribute to therapeutic effects or induce tissue damage 36, 37. Herein, Hep cells showed the highest module score for P450 drug metabolism (Figure 3E). Multi-omics analysis identified P450 genes, such as *Cyp3a11, Cyp2c29, Cyp2b10*, and *Cyp2a5*, as being specifically expressed in Hep (Figure 3F). Notably, RIF treatment increased the expression level of *Cyp3a11* through the indirect activation of PXR 10.

### RIF specifically induced zoned damage to hepatocytes

As previously indicated, RIF exposure resulted in a reduction in the cellular composition of Hep (Figure 3D). Consequently, we analyzed DEGs in Hep cells with RIF, revealing significant enrichment in pathways related to fatty acids (Figure S4A). Furthermore, DEGs were enriched in stress-related pathways, for instance responses to oxidative stress and endoplasmic reticulum stress, as well as processes such as ROS metabolism, TNF production, and apoptotic processes in inflammatory cells (Figure S4A; Table S7).

**Figure 4:**
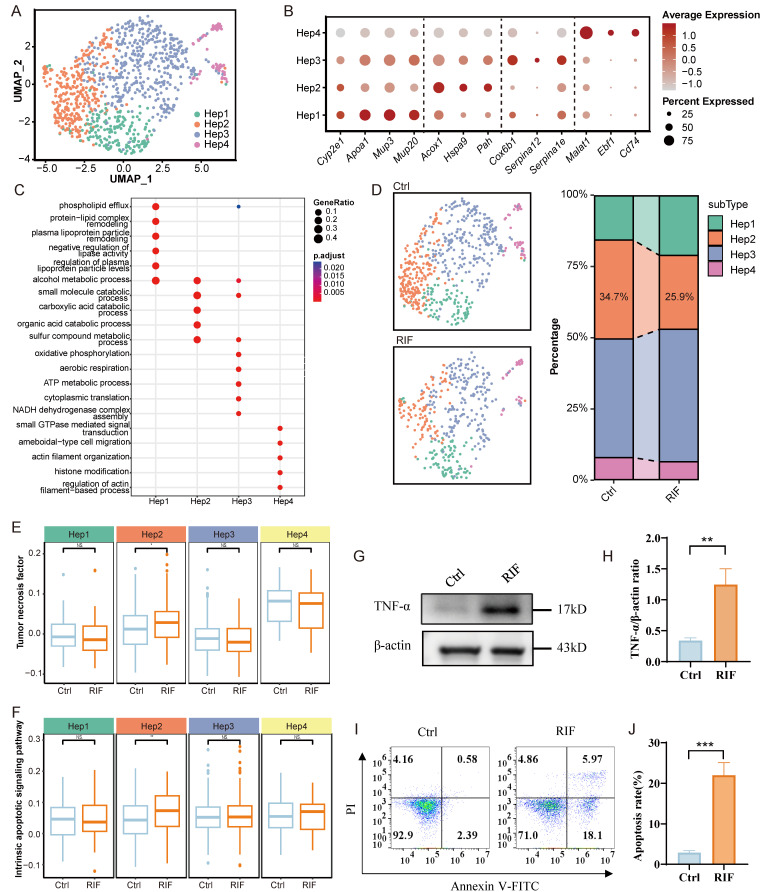
RIF specifically induced zoned damage to hepatocytes. (A) UMAP shows subtypes of hepatocytes. (B-C) Dot plot shows top marker genes, and BP enrichment of cell types associated marker genes in each cell type. (D) Cellular ratio in control and RIF groups. (E-F) Distribution of gene set scores of tumor necrosis factor and intrinsic apoptotic signaling pathway. (G-H) Relative levels of TNF-α in the two groups (** *p* < 0.01). (I-J) RIF-induced apoptosis of hepatocytes (*** *p* < 0.001).

To investigate zoned changes in Hep cells following RIF treatment, we divided Hep cells into four subtypes: Hep1 (*Cyp2e1^+^, Apoa1^+^, Mup3^+^,* and *Mup20^+^*), Hep2 (*Acox1^+^, Hspa9^+^*, and *Pah^+^*), Hep3 (*Cox6b1^+^, Serpina12^+^,* and *Serpina1e^+^*), and Hep4 (*Malat1^+^, Ebf1^+^*, and *Cd74^+^*) (Figure 4A, B; Table S8). Each subtype exhibited a distinct expression profile (Figure S4B). Functional enrichment analysis revealed that markers related to small molecule catabolism and carboxylic acid catabolism were particularly enriched in Hep2. Hep3 was associated with energy metabolism, while Hep1 was linked to lipoprotein metabolism, and Hep4 was associated with cell migration (Figure 4C). Notably, the proportion of Hep2 cells decreased significantly from 34.7% in the control group to 25.9% following RIF treatment, whereas Hep4 showed only a slight reduction. In contrast, the proportions of Hep1 and Hep3 cells increased in response to RIF treatment (Figure 4D).

As displayed in Figure 3E, Hep cells were primary contributors to P450 drug metabolism. We next examined P450 drug metabolism-related gene expression across the four Hep subtypes. Results indicate that the expression levels of P450 genes were significantly elevated in Hep2, Hep3 and Hep4 following RIF exposure (Figure S4C). Of particular interest, Hep2 exhibited the highest expression of P450-related DEGs, such as *Cyp3a11*, suggesting enhanced drug metabolism activity in this subtype under RIF treatment (Figure S4D). Enhanced drug metabolism may generate reactive intermediates, subsequently leading to increased ROS production 38. Consistent with this, Hep2 showed the highest ROS expression levels compared to the other subtypes (Figure S4E). Moreover, Hep2 also exhibited increased expression of gene sets associated with endoplasmic reticulum stress and oxidative stress (Figure S4F-G).

TNF-α is pivotal in inducing mitochondrial ROS production, inflammation, and apoptosis 39-41. Our omics data indeed revealed alterations in the TNF pathway (Figure 2F). We thus assessed the expression of the TNF pathway across the four Hep subtypes and found a significant increase in Hep2 (Figure 4E). Western blot analysis confirmed an increased expression level of TNF-α following RIF exposure (Figure 4G-H). Additionally, the intrinsic apoptotic signaling pathway was upregulated in both Hep2 and Hep4, with Hep2 showing the most pronounced changes, consistent with the observed reduction in cellular proportions of these subtypes (Figure 4D, 4F). Pathway analysis in Hep2 further revealed the activation of oxidative stress-induced intrinsic apoptotic pathway and regulation of epithelial cell apoptotic process in Hep2 (Figure S5A-B). Subsequently, we analyzed the expression levels of apoptosis-related markers, including Casp3, Casp6, Casp7, Bclaf1, Bid, and Bad, and found that these proteins were upregulated following RIF treatment (Figure S5C). Flow cytometry results verified that the apoptosis rate rose with RIF exposure (Figure 4I-J). WB analysis further validated the activation of the pro-apoptotic marker caspase-3 (Figure S5D). Notably, anti-apoptotic protein Bcl-2 was inhibited and the Bcl-2/Bax ratio significantly reduced following RIF exposure (Figure S5E). Furthermore, using pyscenic to explore regulatory networks in Hep cells, we identified several transcription factors (TFs) activated by RIF treatment (Figure S5F). Notably, Hlf and Hif1a, which are involved in pro-inflammatory responses, were among these TFs. In summary, RIF exposure induces zonal damage in Hep cells, with activation of P450 metabolism leading to increased ROS production, inflammation, and apoptosis in specific Hep subtypes.

### RIF induces ROS production and lipid metabolism dysfunction

**Figure 5:**
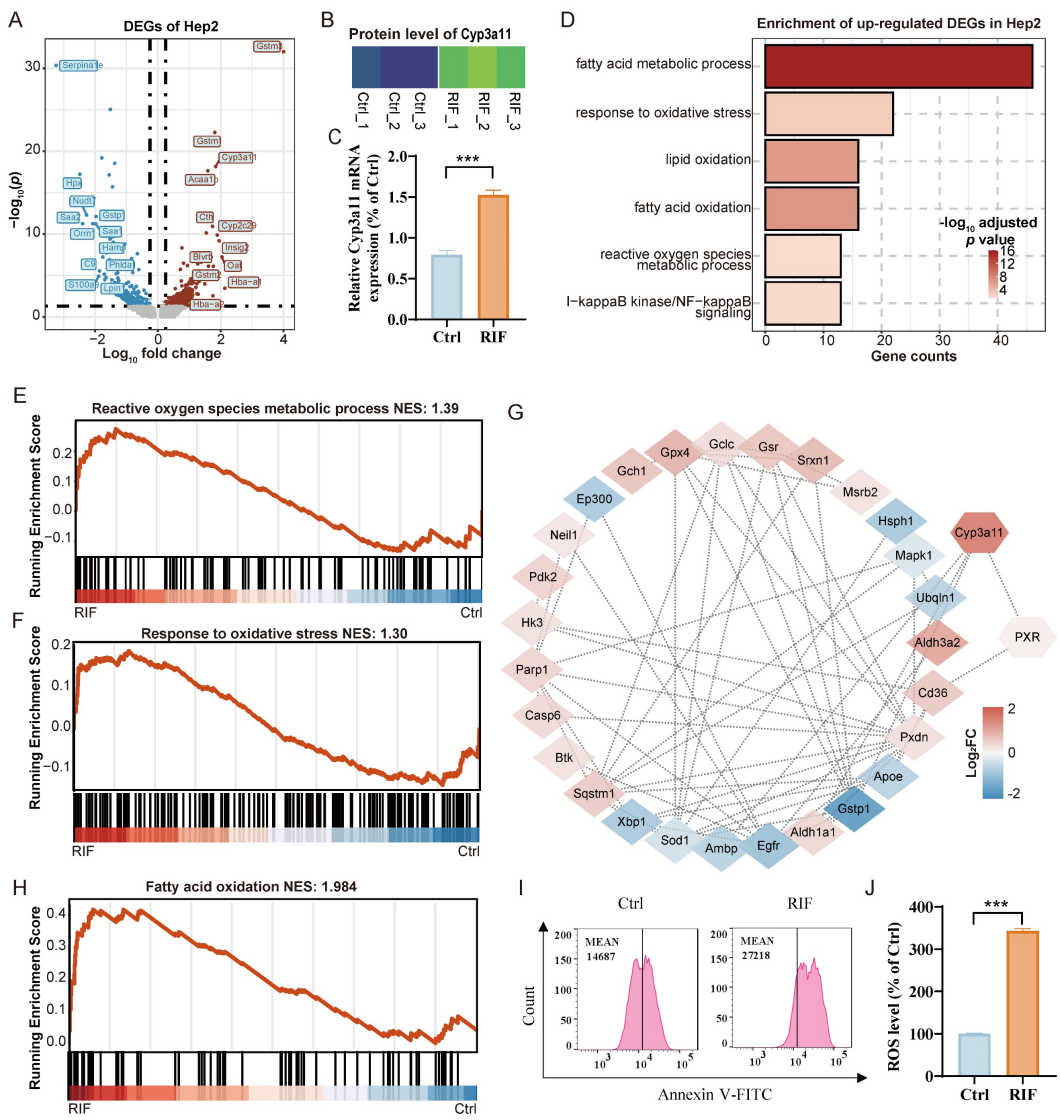
RIF-induced increasing ROS and lipid metabolism dysfunction. (A) The volcano plot displays DEGs in Hep2. Gene symbols represent top 12 up-regulated and down-regulated DEGs. (B) Protein contents of DEGs *Cyp3a11.* (C) Expression level of *Cyp3a11* by qPCR (*** *p* < 0.001). (D) BP enrichment of up-regulated DEGs in Hep2. (E-F) Gene set enrichment analysis (GSEA) of ROS and oxidative stress between control and RIF groups in Hep2. (G) Network of PXR, *Cyp3a11* and oxidative stress associated DEGs in Hep2 (Red gradient represents log_2_ fold change of up-regulated genes and blue gradient represents log_2_ fold change of down-regulated genes). (H) GSEA of fatty acid oxidation in two groups. (I-J) Flow cytometry detection of ROS levels in control and RIF groups (*** *p* < 0.001). NES, Normalized Enrichment Score.

To further investigate the molecular mechanisms underlying RIF-induced zonal damage, we reanalyzed the DEGs from our bulk RNA-seq and DEPs from our label-free proteomics datasets. Enrichment analysis revealed significant changes in genes related to fatty acid metabolism, ROS metabolism, oxidative stress responses, xenobiotic metabolic process, and inflammatory responses across both datasets (Figure S6A-B; Table S9-10). Given the severe injury observed in Hep2 cells following RIF exposure, we examined the distribution of DEGs in Hep2 cells, highlighting the top 12 DEGs (Downregulated: *Serpina1e*, *Hpx, Saa2, Nudt7, Orm1, Saa1, Gstp1, S100a9, C9, Hamp, Phlda1* and *Lpin1*; Upregulated: *Gstm3, Hba-a1, Oat, Insig2, Cyp2c29, Cyp3a11, Gstm1, Gstm2, Hba-a2, Cth, Blvrb* and *Acaa1b*) (Figure 5A). In line with expression levels in Hep2, protein levels of *Cyp3a11* increased following RIF exposure (Figure 5B), and qPCR results further confirmed an increased expression of *Cyp3a11* (Figure 5C).

Further biological process enrichment analysis of upregulated DEGs in Hep2 revealed that lipid oxidation, oxidative stress responses, and ROS metabolism was significantly enriched, consistent with our findings from both the bulk RNA-seq and label-free proteomics datasets (Figure 5D; Figure S6A-B), indicating that these pathways are integral to RIF-induced hepatotoxicity. Interestingly, RIF specifically activated the expression of PXR in Hep2 cells, leading to the upregulation of PXR target genes such as *Cyp3a11* (Figure S6C). As a PXR target gene, *Cyp3a11* serves as a key enzyme in RIF metabolism. Previous research has indicated that RIF-induced ER stress and ROS are crucial in hepatotoxicity 42. Herein, gene set enrichment analysis (GSEA) confirmed the activation of ROS metabolism and oxidative stress responses (Figure 5E-F). Overall, Hep2 cells exhibited strong P450 drug metabolism and ROS production upon RIF induction, and the process potentially involved the activation of PXR and *Cyp3a11*.

To assess the relationship between *Cyp3a11* and PXR with DEGs involved in ROS and oxidative stress in Hep2 cells, we analyzed the expression of related DEGs and their regulatory network. Results indicated that PXR directly regulated *Cd36* expression and indirectly activated *Cyp3a11*, which contributed to reduce *Gstp1* expression. Concurrently, the levels of *Sod1, Xbp1* and *Egfr* decreased (Figure 5G), while apoptotic genes such as *Btk, Pxdn, Casp6, Parp1* and *Gclc* were upregulated (Figure 5G). Oxidative stress arises from overproduction of ROS that surpasses antioxidant capacity, which can damage biological macromolecules, leading to DNA base oxidation, lipid peroxidation and protein carbonylation. Our omics data suggest that lipid metabolism dysfunction plays a pivotal role in hepatotoxicity induced by RIF. Thus, we assessed lipid oxidation and fatty acid oxidation, finding increased expression levels of these pathways, particularly in Hep2 and Hep3 (Figure S6D-E).

Further, to explore the effects of RIF-induced ROS and oxidative stress on fatty acid oxidation, we conducted similar network analyses as above. Changes in gene expression, including *Cd36, Pxdn, Pdk2, Parp1, Sod1*, *Egfr*, and *Xbp1*, contributed to the downregulation of mTOR (Figure S6F). Previous studies have shown that mTOR inhibition accelerates β-oxidation and increases the catabolism of free fatty acids 43. Furthermore, upregulated genes such as *Acox1* and *Acaa1b* encode enzymes involved in very-long-chain fatty acid β-oxidation, with *Acaa1b* playing a significant role in peroxisomal β-oxidation 44. GSEA analysis further confirmed the activation of fatty acid oxidation (Figure 5H). Importantly, consistent with the findings from scRNA sequencing, flow cytometry results indicated elevated ROS levels following RIF exposure (Figure 5I-J). As mitochondria are the major source of ROS, we investigated whether RIF induced dysfunction of mitochondrial functions. Results indicate that RIF treatment disrupts key mitochondrial processes, including respiratory chain assembly and transport, the tricarboxylic acid cycle, and oxidative phosphorylation in Hep2 (Figure S7A-B). *In vitro* Cellular models also suggested an increase in mitoROS following RIF treatment (Figure S7C-D).

In summary, RIF exposure induces ROS and oxidative stress, leading to lipid metabolism dysfunction through the PXR/*Cyp3a11* axis.

### Hep recruits macrophages under RIF exposure to further aggravate hepatocyte injury

**Figure 6:**
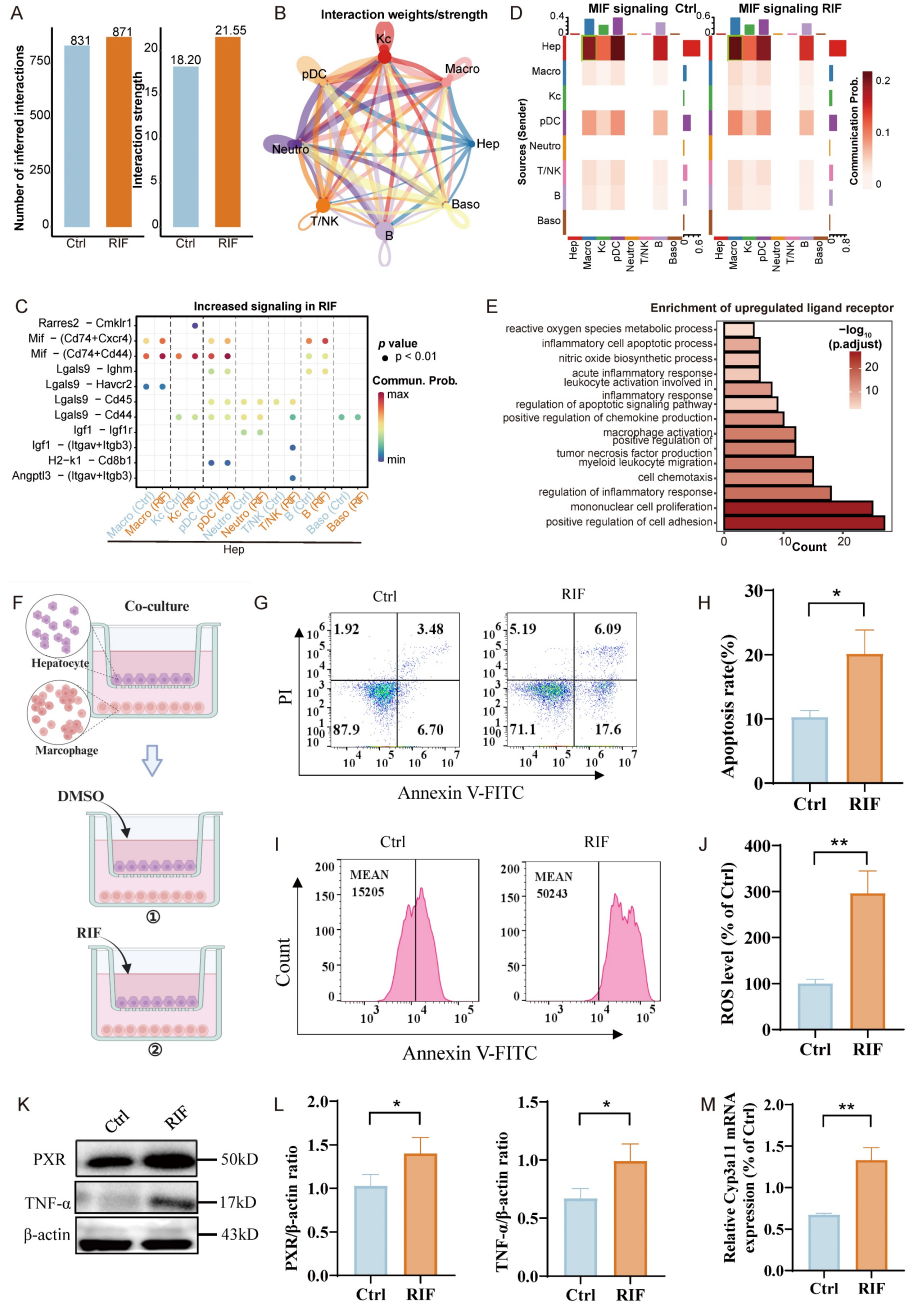
Hep recruiting macrophages to further aggravate hepatocytes injury. (A) The number of intercellular crosstalk inferred interactions (left) and interaction strength (right) in control and RIF groups. (B) The chordal graph shows cell to cell crosstalk between Hep cells and immune cells, colored basing on cell type and thickness degree. (C) The bubble plot represents the communication probability of increasing ligand-receptor pairs between Hep and immune cells. (D) Communication probability of Mif signaling in cellular crosstalk between hepatocytes and immune cells. (E) BP enrichment of up-regulated ligan-receptors in the crosstalk. (F) Co-culture system experimental design process. (G-H) RIF-induced the level of apoptosis and quantitative metrics. (I-J) Flow cytometry detection of ROS levels and quantitative metrics in co-culture system control and RIF groups. (K-L) The protein levels of PXR and TNF-α detected by WB and quantitative statistics. (M) The expression level of *Cyp3a11* measured by qPCR in the co-culture system (* *p* < 0.05; ** *p* < 0.01).

To investigate the influence of immune cells on hepatocytes upon RIF exposure, we conducted a comprehensive crosstalk analysis. Our findings indicated that RIF exposure enhanced the interaction intensity between hepatocytes and immune cells compared to the control (Figure 6A). Notably, we observed substantial interactions between hepatocytes and various immune cell types, including Macro, pDCs and B cells (Figure 6B).

Next, we examined the signaling dynamics in the RIF group, revealing that RIF strengthened the communication probabilities of the ligand-receptor pairs Mif-(Cd74+Cxcr4) and Mif-(Cd74+Cd44) in the crosstalk between hepatocytes and Macro or pDCs (Figure 6C). Additionally, as illustrated in Figure 3D, the proportion of macrophages increased significantly from 46.6% to 53.4% following RIF exposure. This prompted a focused analysis of ligand-receptor interactions between macrophages and hepatocytes, which indicated that Mif signaling from hepatocytes plays a central role in mediating this crosstalk, particularly under RIF exposure (Figure 6D; Figure S8A-B). While Mif is traditionally recognized for inhibiting macrophage migration, it also facilitates the directed migration and recruitment of leukocytes to sites of infection and inflammation 45. We then evaluated the expression of ligands and receptors involved in cellular crosstalk, and performed enrichment analysis of the upregulated ligands and receptors. The results revealed significant enrichment in cell migration pathways, including positive regulation of cell adhesion, myeloid leukocyte migration and chemokine production. Inflammatory-related terms, such as positive regulation of tumor necrosis factor production and acute inflammatory response, were also notably enriched (Figure 6E; Table S11).

Additionally, the upregulated ligand-receptor interactions were associated with the regulation of apoptotic signaling pathways, inflammatory cell apoptosis, and ROS metabolism. Given that upregulated ligand receptors are primarily implicated in inflammation-related pathways, we assessed the expression of inflammation-related ligands and receptors in macrophages. As anticipated, *Mif* expression showed an upward trend, while inflammatory factors *Ccl3, Ccl4, Ccl5*, and *Tnfrsf1a* also exhibited increased expression (Figure S8C). In addition, pro-inflammatory genes, such as *Il1β, Cd68*, and *Ccr5*, exhibited significant upregulation in macrophages (Figure S8D). To further investigate the polarization state of macrophages, we classified them into three subgroups: LCM1, LCM2, and LCM3. LCM1 exhibited specific expression of pro-inflammatory genes, including *Cd86, Cd68,* and *Il1b* (Figure S9A-B). Analysis of macrophage polarization based on M1 and M2 marker genes revealed that LCM1 predominantly exhibited M1 polarization, LCM3 showed a slight tendency toward M1 polarization, while LCM2 was inclined toward M2 polarization (Figure S9C). Cell pseudo-time analysis demonstrated the impact of RIF exposure on macrophage state transitions (Figure S9D). LCM2 cells were primarily positioned at the initial stage of the pseudo-time trajectory and progressively transitioned toward an M1-polarized state. Pseudo-time trajectory branch expression analysis identified DEGs along the trajectory. During the transition to fate 1 (primarily composed of LCM3), upregulated genes were mainly associated with antigen presentation and metabolism-related processes. In contrast, during the transition to fate 2 (primarily composed of LCM1), upregulated genes were predominantly enriched in pathways related to myeloid cell migration, inflammatory cytokine production, and the TNF family (Figure S9E). These findings suggest that RIF exposure drives macrophages toward an M1-polarized state.

In order to further explore the impact of macrophages on hepatocytes, we conducted a co-culture experiment. The cultures were divided into two groups: one treated with DMSO as a control and the other treated with RIF (Figure 6F). After 24 hours, we observed an increased apoptosis rate (Figure 6G-H) and significantly elevated ROS levels in the RIF-treated group compared to controls (Figure 6I-J). Subsequently, we assessed the expression of PXR and TNF-α, finding that RIF elevated their expression levels in agreement with both our silicon-based and experimental data above (Figure 6K-L). Additionally, qPCR results also indicated an increase in *Cyp3a11* expression in the co-culture system (Figure 6M).

In summary, the results suggest that hepatocytes may recruit macrophages following RIF exposure, with the Mif signaling pathway playing a role in this process. The macrophages exhibit a tendency toward M1 polarization. During these cellular interactions, inflammation-related pathways are activated, resulting in increased ROS levels and a higher number of apoptotic cells.

### Rosmarinic acid ameliorates ROS levels and hepatocyte apoptosis

**Figure 7:**
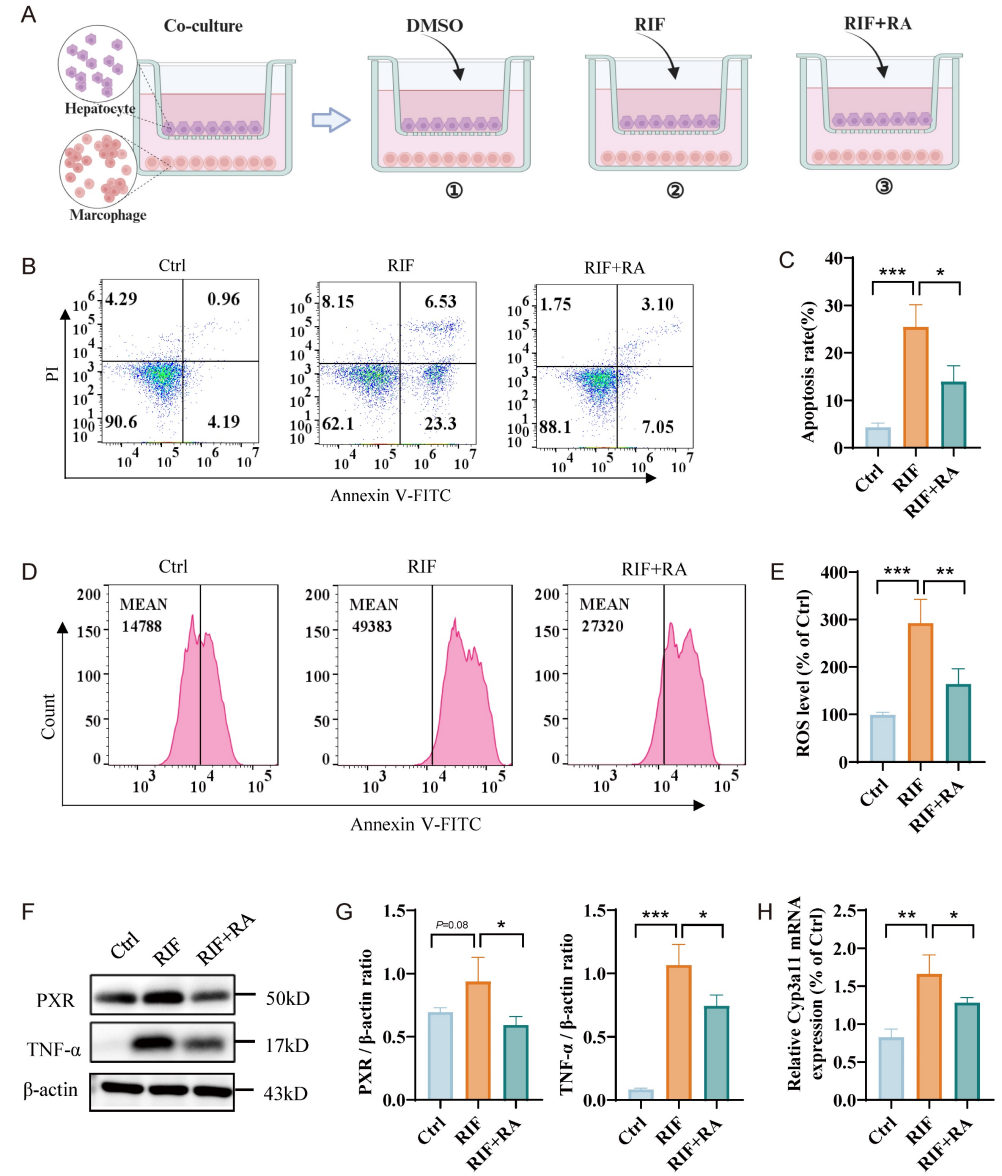
Rosmarinic acid ameliorating ROS level and hepatocytes apoptosis. (A) Schematic diagram of co-culture system: one with DMSO as a control, one with RIF alone, and another with both RIF and RA. (B-C) Flow cytometry apoptosis ratio detection in three groups and quantitative metrics. (D-E) ROS level of the cells with treatment of DMSO, RIF and RIF+RA and quantitative statistics. (F-G) The protein levels of PXR and TNF-a measured by WB and quantitative information. (H) The expression level of *Cyp3a11* measured by qPCR. (* *p* < 0.05; ** *p* < 0.01; *** *p* < 0.001).

Rosmarinic acid (RA) is a natural polyphenol extract that effectively scavenges ROS, alleviating oxidative stress and protecting against drug-induced liver damage. Our results indicate that RIF significantly increases ROS levels and corresponding cellular apoptosis rates. To explore the effect of RA on RIF-induced liver damage, we established three co-culture systems: one with DMSO as a control, one with RIF alone, and another with both RIF and RA (Figure 7A). Consistent with previous findings, RIF treatment caused a significant increase in apoptosis rates (Figure 7B-C) and elevated ROS levels (Figure 7D-E). Notably, in the co-culture treated with RA, ROS levels and apoptosis rates were significantly reduced. Furthermore, RA markedly decreased the expression levels of PXR and TNF-α (Figure 7F-G), as well as the main metabolic enzyme *Cyp3a11* associated with RIF (Figure 7H). Together, these results suggest that RA mitigates RIF-induced ROS and liver cell apoptosis, indicating its potential as a protective agent against RIF-induced hepatotoxicity.

## Discussion

**Figure 8:**
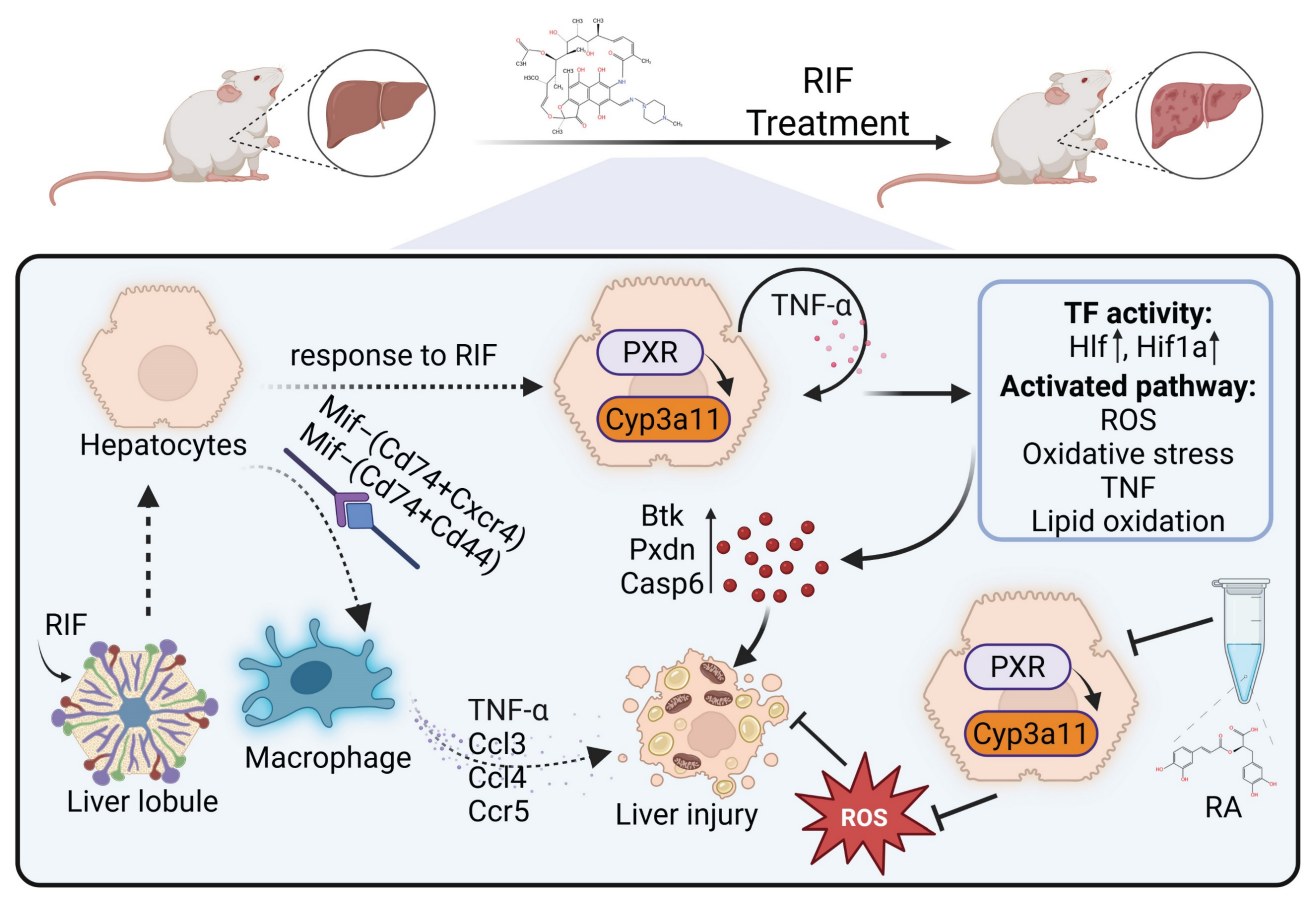
Schematic summary of the mechanism underlying RIF-induced hepatotoxicity and the ameliorative effects of RA. RIF exposure activates PXR and its target gene *Cyp3a11*, enhancing RIF metabolism and increasing ROS production, leading to oxidative stress. Elevated TNF-α levels further raise ROS. Accumulation of ROS enhances lipid oxidation and induces lipid peroxidation, which results in hepatocyte apoptosis. Macrophage recruitment triggers a pro-inflammatory response, worsening hepatocyte injury. In contrast, RA inhibits ROS levels and mitigates this damage.

RIF, a primary anti-TB drug, has been associated with potential hepatotoxicity in several studies 46, 47. We employed multi-omics to comprehensively analyze the mechanism of RIF-induced hepatotoxicity and the mitigative effects of RA (Figure 8). At the single-cell level, we identified a specific region (Hep2) of RIF-induced hepatocyte injury, primarily involved in small molecule and carboxylic acid catabolism. Under RIF exposure, ROS metabolism and oxidative stress in this region were enhanced, alongside an increase in fatty acid metabolism. RNA-seq and proteomic analyses confirmed RIF-induced DEGs in related pathways. Differential metabolites, including 12(S)-HETE, prostaglandin E2, palmitic acid and docosapentaenoic acid, were associated with fatty acid peroxidation 48-51. While our findings confirmed the activation of fatty acid-related metabolic pathways, additional studies are required to establish whether the RIF-induced increase in ROS directly contributes to lipid peroxidation.

Nevertheless, our multi-omics data and ROS experimental results indicate that ROS metabolic activation plays a central role in RIF-induced hepatotoxicity. ER stress, which is closely associated with elevated ROS levels, has been implicated in RIF-induced hepatotoxicity 42, 52. Our results confirmed ER stress activation upon RIF treatment. Furthermore, we observed a significant enhancement of endogenous apoptotic signaling in Hep2 following RIF treatment, with an expanded expression range of apoptosis-related genes. These findings align with previous reports of RIF-induced hepatocyte apoptosis 15, 53. As the major source of ROS, mitochondrial function was disrupted by RIF treatment. Specifically, RIF impaired the assembly of the electron transport chain and electron transport complex. The mitochondrial functional pathways of oxidative phosphorylation and tricarboxylic acid cycle were inhibited by RIF, suggesting that mitochondria play a crucial role in the elevated ROS levels. Additionally, our cellular experiments indicated an increase of mitoROS.

Anti-TB drugs metabolized by P450 enzymes generate toxic intermediates that contribute to ROS production 13. Elevated ROS levels initiate lipid peroxidation (LPO), a key factor in hepatocellular injury 54-56. Studies have established a strong correlation between oxidative stress and anti-TB drug-induced hepatotoxicity, where excessive ROS accumulation surpasses the cellular antioxidant capacity 57, 58. Our single-cell analysis revealed that hepatocytes exhibited the highest P450 pathway activity comparing to other cell types. P450 genes such as *Cyp3a11, Cyp2a5, Cyp2c29*, and *Cyp2b10*, were differentially expressed in both the RNA-seq and proteomic analyses. The expression of these genes was especially increased in Hep2. As an inducer of PXR, RIF activated PXR expression in Hep2 cells, leading to the upregulation of *Cyp3a11*. The coordinated changes in PXR and *Cyp3a11* resulted in a significant downregulation of antioxidant genes, such as *Sod1, Xbp1* and *Gstp1*, while promoting the upregulation of apoptosis-related genes, including *Pxdn, Btk, Casp6, Parp1* and *Gclc*. The regulatory hub gene mTOR encodes a protein kinase that governs cellular metabolism, catabolism, immune responses, and migration to maintain homeostasis 59. Notably, mTOR negatively regulates fatty acid oxidation, and its inhibition has been shown to enhance this process 60. In our study, RIF suppressed mTOR expression while concurrently activating Acox1 and Acaa1b, key proteins involved in very-long-chain fatty acid β-oxidation. These findings suggest a mechanistic pathway underlying RIF-induced liver injury. Drug metabolism generally involves phase I and phase II reactions 61, 62. Our study specifically identified alterations in P450-related phase I metabolic enzymes, while no significant changes were detected in phase II metabolic enzymes.

Liver macrophages are crucial for maintaining hepatic homeostasis. During inflammatory states, they become activated and influence hepatocyte fate 63, 64. Hepatocytes can recruit pro-inflammatory immune cells, contributing to immune responses 65. An increase in macrophages is correlated with the extent of liver injury 66, 67. Our H&E staining results indicated the infiltration of inflammatory cells, and an increasing cellular ratio of Macro was observed upon RIF exposure in the scRNA-seq data. The cellular crosstalk results suggest that hepatocytes recruited macrophages to the damage site. Macrophages exhibit remarkable plasticity and can polarize into pro-inflammatory M1 or anti-inflammatory M2 phenotypes in response to specific stimuli 68, 69. In our study, recruited macrophages exhibited activation of pro-inflammatory genes and a tendency toward M1 polarization. In the hepatocyte-macrophage co-culture model, increased levels of ROS were observed, along with cellular apoptosis induced by increased ROS levels and an increased TNF-α level. These findings suggest that hepatocytes recruit macrophages, exacerbating damage upon RIF exposure. As both our multi-omics data and co-culture experiments indicate increased ROS levels after RIF exposure, the effective removal of ROS and alleviation of oxidative stress may thus be a viable strategy to mitigate RIF-induced hepatotoxicity. In this study, RA treatment effectively reduced ROS levels and hepatocyte apoptosis induced by RIF, suggesting its potential as a therapeutic option for anti-TB drug-induced hepatotoxicity.

## Conclusions

In conclusion, this study provides a comprehensive analysis of the mechanisms involved in RIF-induced hepatotoxicity using a multi-omics approach. The findings reveal that RIF exposure significantly elevates levels of ROS and oxidative stress, leading to localized hepatocellular damage. Moreover, interactions between hepatocytes and macrophages exacerbate the extent of liver injury. Importantly, the natural compound RA demonstrates a protective effect by mitigating ROS levels and reducing liver damage induced by RIF. This research enhances our understanding of RIF-induced hepatotoxicity and highlights the therapeutic potential of RA. The multi-dimensional data generated offer valuable insights and resources for future investigations into the detailed mechanisms underlying RIF-induced liver injury.

## Supplementary Material

Supplementary figures.

Supplementary tables.

## Abbreviations

TB: tuberculosis
ROS: reactive oxygen species
RIF: Rifampicin
RA: Rosmarinic acid
DEGs: differentially expressed genes
UMAP: uniform manifold approximation and projection
PCA: principal component analysis
LC-MS/MS: liquid chromatography-mass spectrometry
WB: western blotting
H&E: Hematoxylin and eosin
RNA-seq: RNA sequencing
Ortho PLS-DA: orthogonal partial least squares-discriminant analysis
Mif: macrophage migration inhibitory factor
Gstp1: glutathione S-transferase pi 1
PXR: pregnane X receptor
TNF-α: tumor necrosis factor α
