# Supplementary material for: Integrated single-cell transcriptomics and proteomics elucidate the molecular mechanisms and detoxification strategy of rifampicin-induced hepatotoxicity
Source: Int J Biol Sci. 2026 Jan 1;22(1):481–99. doi: 10.7150/ijbs.109757 (PMC12681935; doi:10.7150/ijbs.109757)
Supplement: Supplementary file 1 — Supplementary figures. [file ijbsv22p0481s1.pdf]

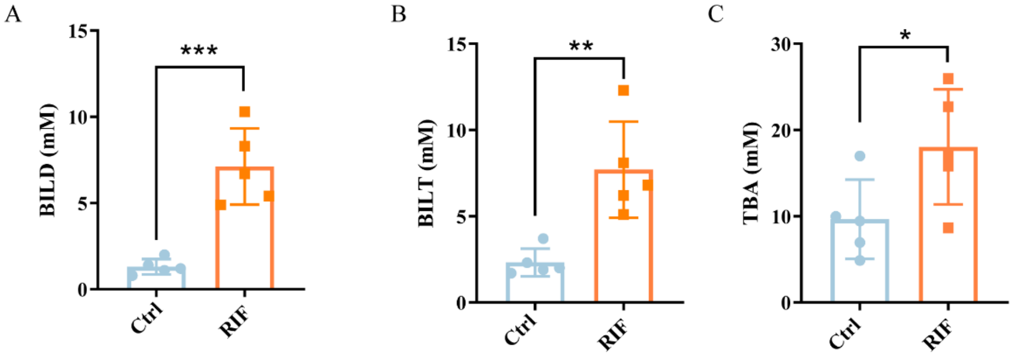

Figure S1 RIF triggered liver toxicity and dysfunction in mice**.** (A-C) Effects of RIF on the levels of serum BILD, BILT and TBA in the indicated groups (n=5, * $p < 0.05$, ** $p < 0.01$, *** $p < 0.001$).

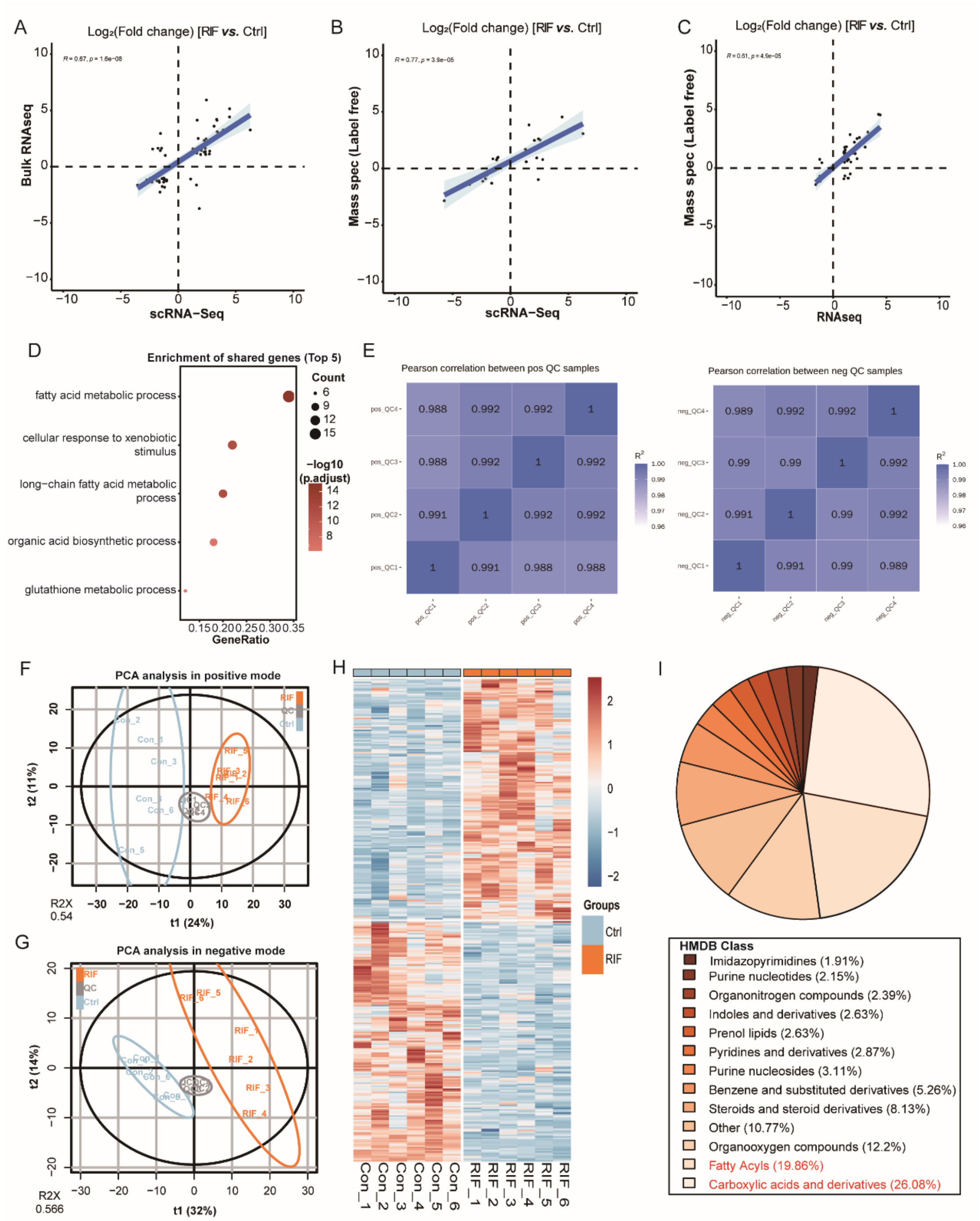

Figure S2 RIF-induced hepatotoxicity involving metabolism dysfunction. (A-C) The scatter plots display correlation of $\log_2$ fold change of DEGs and DEPs between pseudo bulk data of scRNA and bulk RNA-seq (A), pseudo bulk data of scRNA and protein dataset (B), bulk RNA-seq and protein dataset (C). (D) Top 5 of BP enrichments for shared gene alterations across the three datasets. (E) Correlation of quality control samples in positive mode (left) and negative mode (right). (F-G) PCA analysis of metabolites of positive mode (F) and negative mode (G). (H) Expression patterns of

differential metabolites samples. (I) Classification statistics of HMDB database annotations in the liver upon RIF treatment.

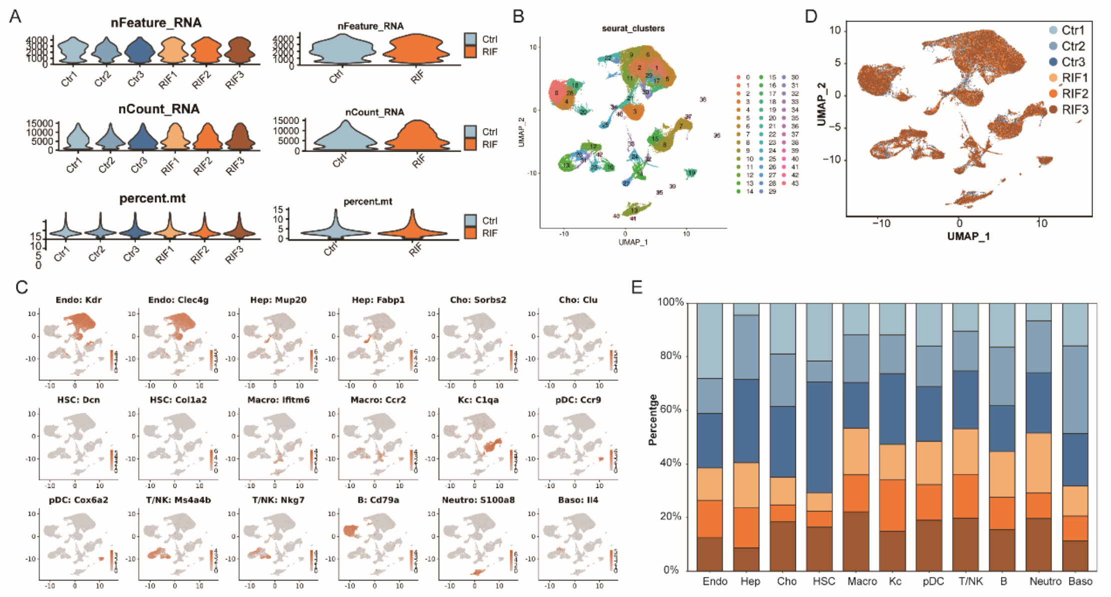

Figure S3 Quality control of scRNA data, marker genes distribution and cellular composition. (A) The number of genes (top), count of transcripts (middle) and percentage of mitochondrial genes (bottom) in each sample and group following quality control. (B) UMAP shows original UMAP clusters. (C) Distribution of classic markers in UMAP. (D-E) UMAP shows the sample distribution (D) and bar plot displays samples composition in each cell type (E).

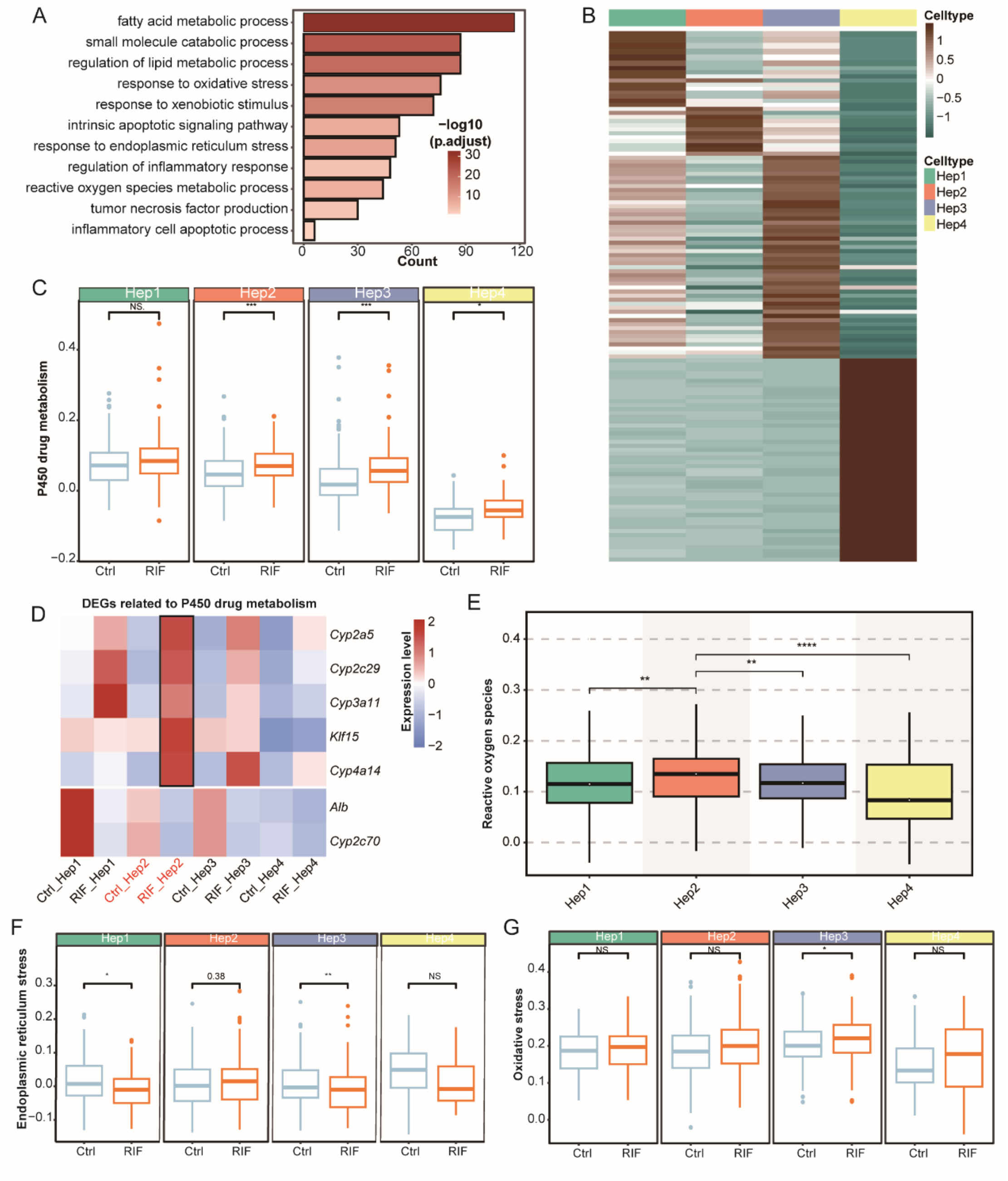

Figure S4 Analysis of the regionally heterogeneous response of hepatocytes. (A) Top BP enrichment of DEGs in hepatocytes. (B) Expression patterns of marker genes for each subtype. (C) Distribution of expression level of P450 drug metabolism in certain sup type. (D) Heatmap displays expression level of P450 drug metabolism associated DEGs across subtypes. (E) Expression levels of gene set reactive oxygen species (E). (F-G) The pathway comparisons of the endoplasmic reticulum stress (F) and oxidative stress (G) scores across four sub types. (* $p < 0.05$, ** $p < 0.01$, *** $p < 0.001$).

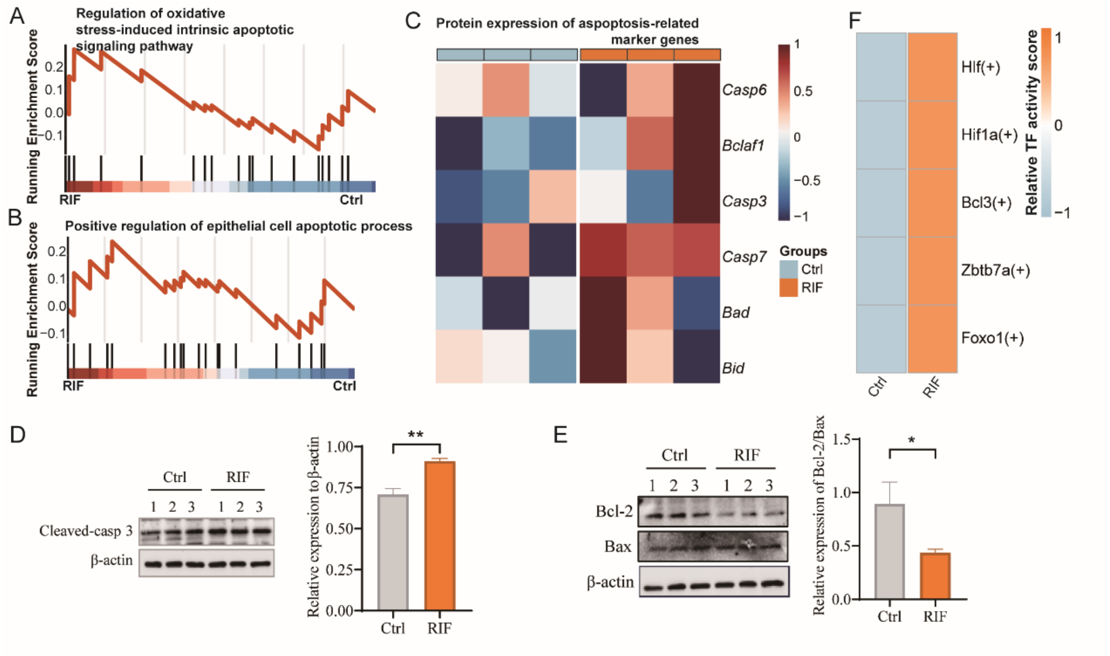

Figure S5 RIF-induced hepatocyte apoptosis. (A-B) GSEA distribution of regulation of oxidative stress-induced intrinsic apoptotic signaling pathway and regulation of epithelial cell apoptotic process in Hep2. (C) Protein expression of pro-apoptotic marker genes in samples of RIF and Ctrl groups. (D) Relative levels of Caspase-3 in the two groups (** $p < 0.01$). (E) Relative levels of Bcl-2 and Bax, and ratio of Bcl-2/Bax in the two groups (* $p < 0.05$). (F) Heatmap displays the increasing relative activity score of transcription factors following RIF treatment.

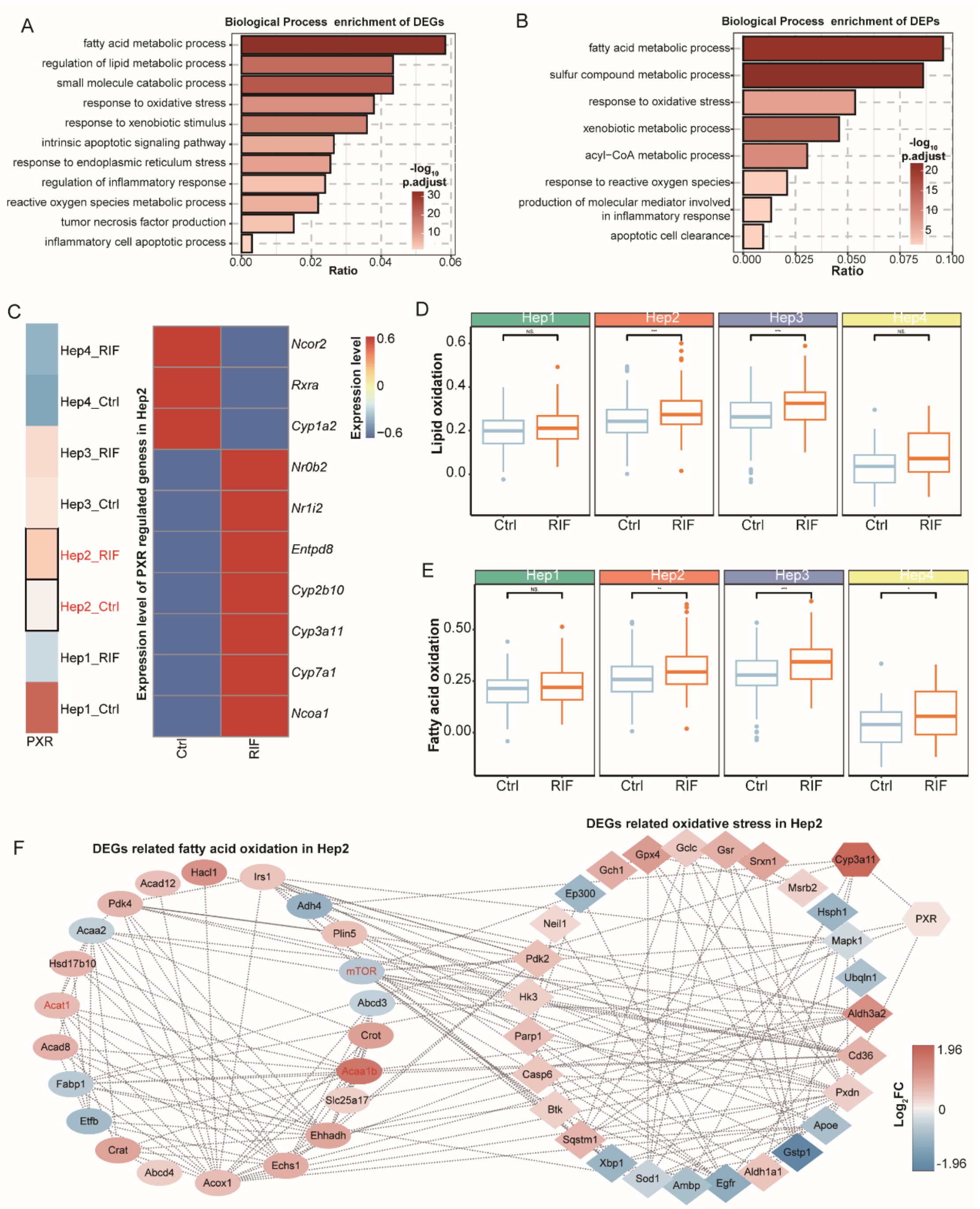

Figure S6 RIF-induced increasing ROS and lipid metabolism dysfunction. (A-B) Biological process enrichment of DEGs of bulk RNA-seq and DEPs of mass spec proteomics. (C) Expression level of PXR in each subtype upon RIF exposure (left) and expression of targeted genes of PXR in Hep2 under RIF treatment (right). (D-E) Expression level of gene set lipid oxidation and fatty acid oxidation across four subtypes upon RIF treatment (* $p < 0.05$, ** $p < 0.01$, *** $p < 0.001$). (F) The network illustrates the correlation between PXR, Cyp3a11, and DEGs involved in ROS and oxidative stress and fatty acid oxidation in Hep2 cells.

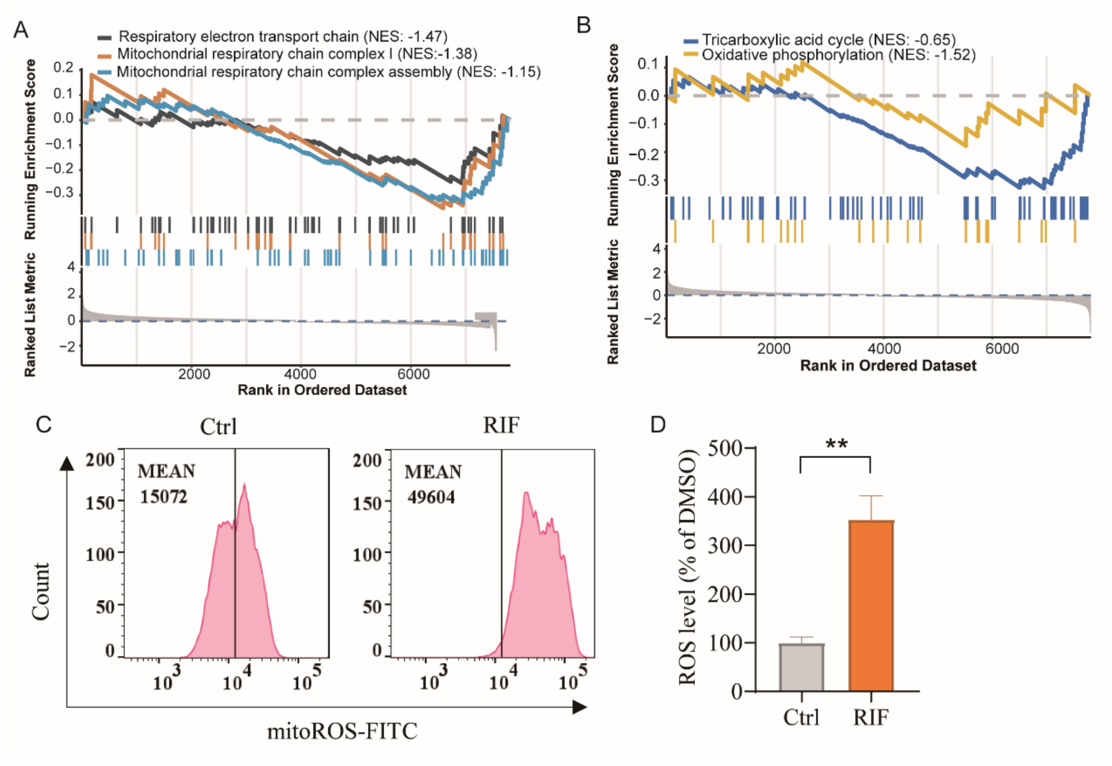

Figure S7 RIF inducing mitochondrial dysfunction. (A-B) GSEA result of mitochondrial respiratory chain (A) and the mitochondrial functional pathways of oxidative phosphorylation and tricarboxylic acid cycle (B). (C-D) flow cytometry detection of mitoROS levels (C) and quantitative metrics in control and RIF groups (D) (** $p < 0.01$).

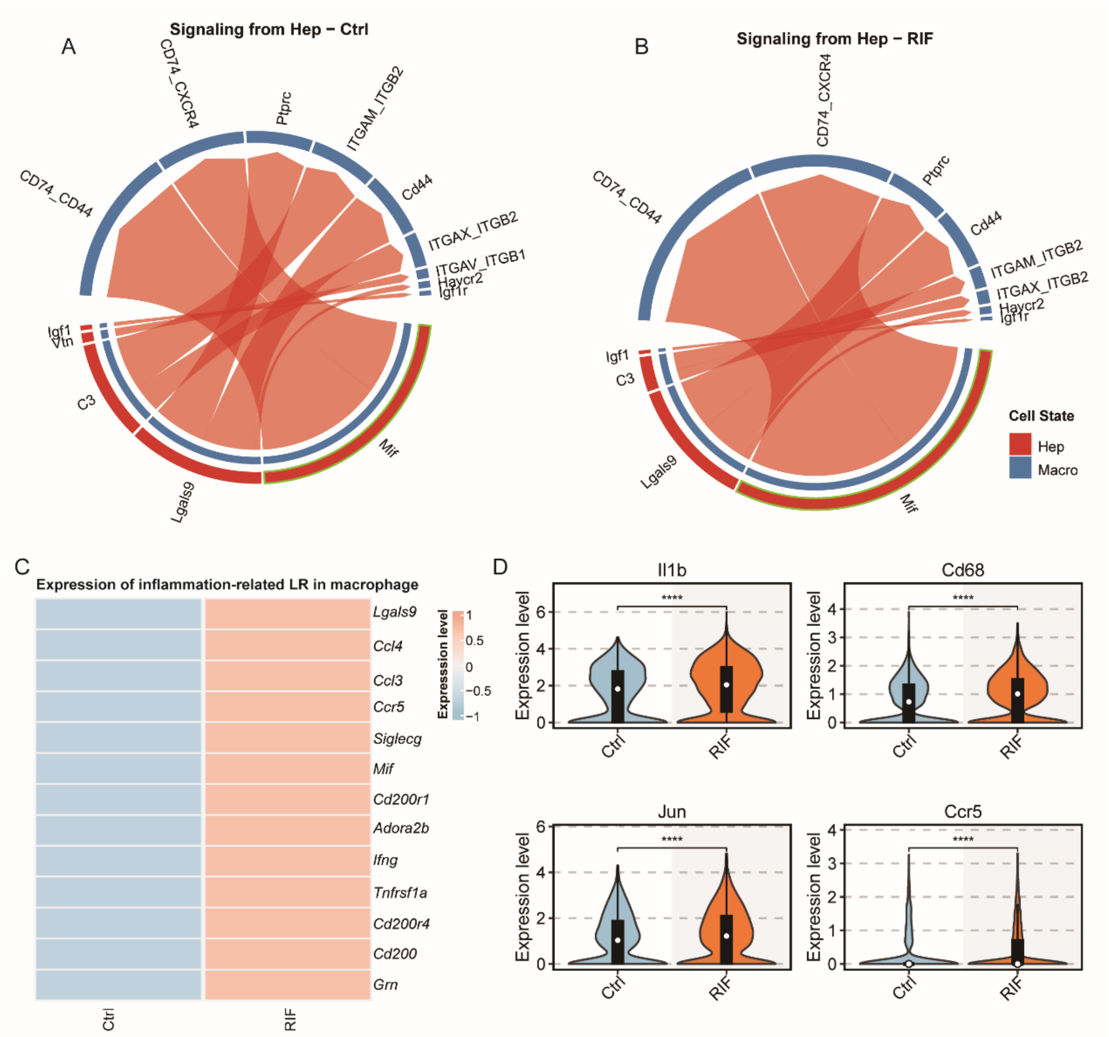

Figure S8 Cellular crosstalk between hepatocytes and macrophage. (A-B) Ligand-receptors involving signaling sent from hepatocytes to macrophage in control group and RIF treatment group. (C) Expression levels of upregulated ligand-receptors in macrophages involved in inflammation. (D) Comparison of pro-inflammatory gene expression in macrophage between the Ctrl and RIF groups (*** $p < 0.001$).

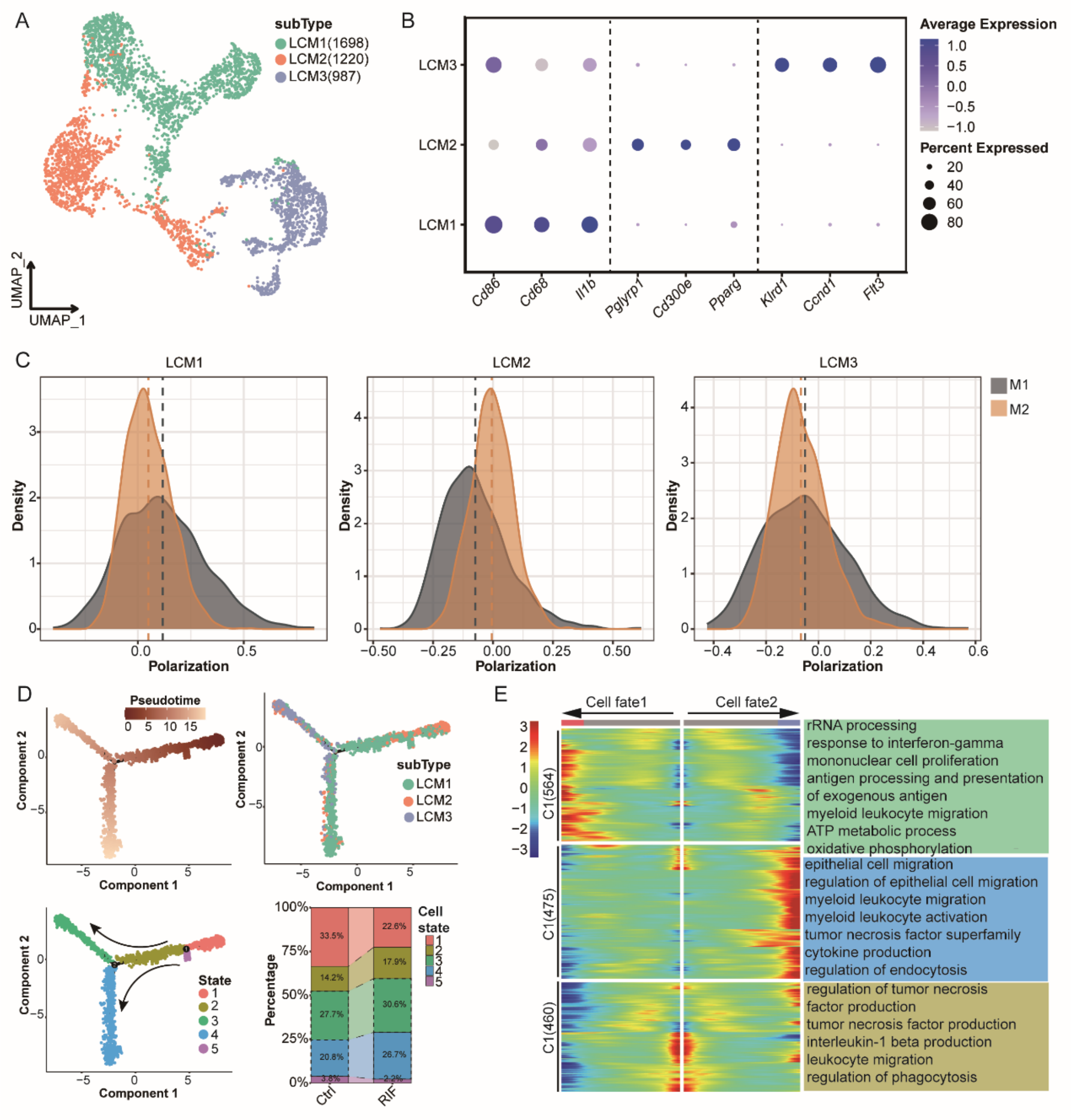

Figure S9 RIF exposure drives macrophages toward an M1-polarized state. (A) UMAP shows subtypes of Macrophage. (B) Dot plot shows top marker genes of certain subtype. (C) The polarization ability of macrophage subtypes. (D) Pseudotime trajectory of macrophages (top left), distribution of macrophage sub types (top right), cellular state (bottom left) along the trajectory and statistical analysis of macrophage cell states in Ctrl and RIF groups (bottom right). (E) Heatmap displaying lineage-dependent expression patterns of branch-specific genes (right) and the corresponding upregulated GO-enriched terms for each cluster (left).
