# Supplementary material for: Integrated single-cell transcriptomics and proteomics elucidate the molecular mechanisms and detoxification strategy of rifampicin-induced hepatotoxicity
Source: Int J Biol Sci. 2026 Jan 1;22(1):481–99. doi: 10.7150/ijbs.109757 (PMC12681935; doi:10.7150/ijbs.109757)
Supplement: Supplementary file 2 — Supplementary tables. [file ijbsv22p0481s2.zip › Supplementary_materials_tables/Supplementary MaterialS4_5.pdf]

Table S4 Quality control of single cell sequencing upon RIF exposure

**Sample**	**nFeature**	**nCount**	**percent.mt (%)**	**Clean Cell**	**Feature (median)**
Ctr1	200-4,500	500-15,000	15	13,172	2,389
Ctr2	200-4,500	500-15,000	15	10,628	2,011
Ctr3	200-4,500	500-15,000	15	13,976	2,079
RIF1	200-4,500	500-15,000	15	9,881	2,064
RIF2	200-4,500	500-15,000	15	9,589	2,183
RIF3	200-4,500	500-15,000	15	10,210	2,083

Note: Control sample group: Ctr1, Ctr2, Ctr3; Treatment sample group: RIF1, RIF2, RIF3. nFeature: Range of gene numbers in the cells of each sample; nCount: Range of unique molecular identifier (UMI) counts in the cells of each sample; percent.mt: Percentage of mitochondrial content, with a cutoff of < 15% in the cells of each sample.

Table S5 qPCR primer sequences (Cyp3a11)

FP:	RP:
ACCTGGGTGCTCCTAGCAATC	AATCCCGCCGGTTTGTGAAG
